# Exosome-like nanoparticles derived from *Astragali Radix-Curcumae Rhizoma* co-decoction enhance oral bioavailability and provide synergistic efficacy and reduced toxicity in combination with 5-fluorouracil for lung cancer therapy

**DOI:** 10.1016/j.ijpx.2026.100611

**Published:** 2026-07-13

**Authors:** Ruiyue Fang, Jingbei Zhang, Yiyang Cen, Xufeng Yang, Xiaoping Fang, Ziyang Wu, Chunyu Huang, Junfeng Wu, Yuhua Yang, Zhuowei Li, Long Xi, Yan Ma, Shixia Guan, Liping Cao

**Affiliations:** a School of Pharmaceutical Sciences, Guangzhou University of Chinese Medicine, Guangzhou 510006, China; b The Second Affiliated Hospital of Guangzhou University of Chinese Medicine (The Second Clinical Medical College, Guangzhou University of Chinese Medicine), Guangzhou 510120, China; c Shenzhen Bao'an Traditional Chinese Medicine Hospital, Guangzhou University of Chinese Medicine, Shenzhen, Guangdong 518000, China

**Keywords:** *Astragali Radix-Curcumae Rhizoma* pair-derived exosome-like nanoparticles, Oral bioavailability, Antitumour efficacy, 5-fluorouracil, Reduced toxicity

## Abstract

The low oral bioavailability of bioactive components from the *Astragali Radix-Curcumae Rhizoma* herb pair severely restricts its clinical application in antitumour therapy. To address this issue, we developed a natural drug delivery system using exosome-like nanoparticles (ACELNs) isolated from the co-decoction of *Astragali Radix and Curcumae Rhizoma*, which can effectively encapsulate and preserve the bioactive components from the herbal pair. Systematic characterization confirmed that ACELNs maintained excellent structural integrity, flat spheroid morphology, and favorable gastrointestinal stability with desirable colloidal properties. Cellular and rat pharmacokinetic studies demonstrated that ACELNs significantly improved the oral bioavailability of the loaded active components. In Lewis lung cancer ectopic mouse model, the combination of ACELNs and 5-fluorouracil (5-FU) exhibited superior antitumour efficacy and excellent biocompatibility. This natural nanocarrier strategy provides a promising approach to overcome the bioavailability barrier of traditional Chinese medicine components and achieve synergistic therapeutic effects.

## Graphical abstract

**Figure:**
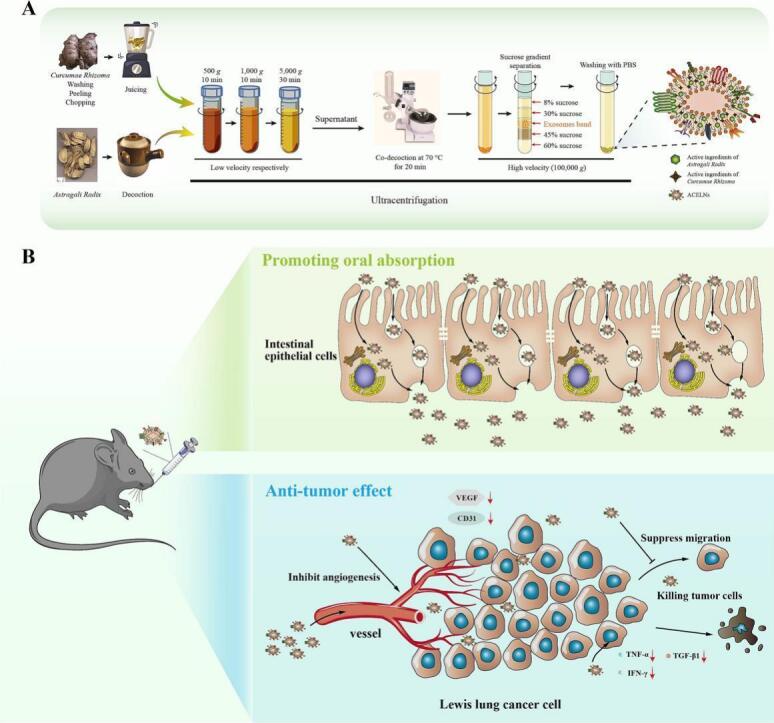

## 1 Introduction

Herbal compatibility represents the core characteristic and clinical advantage of traditional Chinese medicine (TCM), which achieves synergistic efficacy and toxicity reduction through multi-component and multi-target therapeutic effects (Bian et al., 2022; Liang et al., 2025; Wang et al., 2025b). Pharmacokinetic and serum pharmacochemical studies have confirmed that herbal compatibility significantly improves the in vivo absorption of poorly soluble bioactive components (Wu et al., 2023; Yang et al., 2026). However, the mechanisms underlying the absorption, distribution, metabolism and synergistic effects of these components remain incompletely elucidated, which severely restricts the modernization and clinical translation of classic TCM herbal pairs (Wang et al., 2026).

*Astragali Radix-Curcumae Rhizoma* is a classic herbal pair widely used in clinical antitumor therapy (Liang et al., 2025; Sun et al., 2024; Wang et al., 2025a). Accumulating evidence proves this combination exerts synergistic anti-tumor, anti-inflammatory and gut barrier-protective effects by regulating intestinal microbiota and local immune microenvironment, which cannot be fully achieved by single herb treatment alone (Su et al., 2023). Numerous studies have verified that its combined efficacy is significantly superior to that of single herb. The main bioactive components of *Astragali Radix* include astragaloside IV, flavonoids (calycosin, formononetin), etc., while the core components of *Curcumae Rhizoma* consist of curcumin, curcumol, β-elemene, curdione, etc., all of which exert definite antitumor activities (Chen et al., 2021; Guo et al., 2019; Yin et al., 2018). However, relevant studies on the absorption characteristics of active components from *Astragali Radix* and *Curcumae Rhizoma* have demonstrated that most of their bioactive components are poorly absorbed after oral administration. Specifically, astragaloside IV from *Astragali Radix* exhibits significantly reduced intestinal mucosal permeability due to its low lipophilicity and high molecular weight, making it difficult to be effectively taken up by intestinal epithelial cells (Huang et al., 2006). As reported by biotransformation-related research, the low oral exposure of astragaloside IV largely limits its in vivo pharmacological activity even after intestinal metabolism (Hu et al., 2023). Curcumin from *Curcumae Rhizoma* is limited by poor chemical stability and extremely low water solubility, and is prone to degradation in the gastrointestinal tract after oral administration, ultimately resulting in very low oral bioavailability (Ban et al., 2020; Ma et al., 2019). In addition, volatile components such as furanodiene, curzerene, and β-elemene from *Curcumae Rhizoma* cannot penetrate the Caco-2 cell monolayer model, which simulates the intestinal absorption barrier (Bai et al., 2021). Furthermore, furanodiene and β-elemene show poor stability in physiological fluids, further hindering their efficient absorption in the small intestine (Kim et al., 2019; Pei et al., 2012; Zhai et al., 2018). These inherent defects related to absorption collectively constitute a critical bottleneck restricting the full therapeutic efficacy of the *Astragali Radix-Curcumae Rhizoma* herbal pair (Tay et al., 2019; Wang et al., 2019; Xu et al., 2018).

Recent studies have revealed that plants could spontaneously form nanostructured carriers during decoction, including complexes, micelles and exosome-like nanoparticles (ELNs), which could significantly improve the solubility, membrane transport and in vivo absorption of bioactive components (Li et al., 2019). Plant-derived ELNs have become a research hotspot in the field of nanodelivery systems due to their natural origin, favorable biocompatibility, high colloidal stability and natural drug-loading capacity (Gong et al., 2026; Yang et al., 2025). To date, ELNs with anti-inflammatory and antitumour activities have been successfully isolated from various plants such as *grapefruit*, *ginger*, *ginseng* and *Rhodiola rosea* (Castelli et al., 2023; Duan et al., 2025; Zhu and He, 2023). However, most existing studies focus on ELNs derived from single herbs, and the colloidal properties, in vivo absorption behaviors, transmembrane transport mechanisms and synergistic antitumour effects combined with chemotherapeutic drugs of ELNs formed by co-decoction of classic TCM herbal pairs remain rarely reported, representing a prominent research gap.

**Scheme 1:**
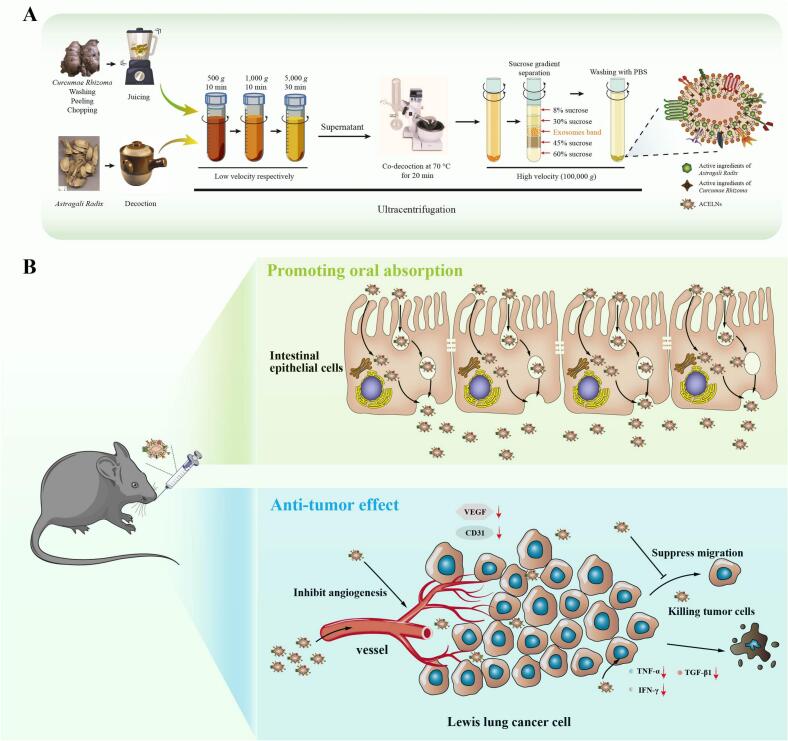
Schematic illustration of preparation procedure and potential mechanism of *Astragali Radix-Curcumae Rhizoma* exosome-like nanoparticles (ACELNs) in promoting bioactive component absorption and enhancing anti-Lewis lung cancer efficacy. All graphical elements of this schematic were independently hand-drawn by the authors using Adobe Illustrator 2024, without any AI generation assistance.

This study constructed *Astragali Radix-Curcumae Rhizoma*-derived exosome-like nanoparticles (ACELNs), which realized efficient encapsulation of bioactive components from the two herbs through co-decoction recombination, and were isolated and purified by ultracentrifugation combined with sucrose density gradient centrifugation (Xu et al., 2026). This study aims to systematically investigate the colloidal properties, gastrointestinal stability, cellular uptake, transmembrane transport and in vivo pharmacokinetic behaviors of ACELNs, and clarify the mechanism of ACELNs in improving the oral bioavailability of bioactive components from *Astragali Radix-Curcumae Rhizoma*. On this basis, the synergistic antitumor effect of ACELNs combined with the clinical chemotherapeutic drug 5-fluorouracil (5-FU) will be further evaluated to reveal the potential value of ACELNs in enhancing chemotherapeutic efficacy and achieving synergistic therapy (Scheme 1). The results of this study will provide experimental evidence for elucidating the scientific connotation of synergistic efficacy of *Astragali Radix-Curcumae Rhizoma* herbal pair, and offer new ideas and strategies for constructing natural and safe TCM-based nanodelivery systems to improve the efficacy of combined tumour therapy.

## 2 Materials and methods

### 2.1 Materials

The materials were presented in the supporting information.

### 2.2 Cells and animals

Caco-2 human colon adenocarcinoma cells and A549 human non-small cell lung cancer cells were obtained from Macao University of Science and Technology (Macao, China). HepG2 human hepatocellular carcinoma cells and Lo2 normal hepatocytes were obtained from Guangzhou University of Chinese Medicine (Guangzhou, China). All cells were grown in DMEM medium with 10% fetal bovine serum (FBS, Nanjing Shenghang Biotechnology Co., Ltd., Nanjing, China), 1% penicillin and streptomycin. The differences were that Caco-2 human colon adenocarcinoma cells was grown in 20% FBS and Lo2 normal hepatocytes was grown in RPMI 1640 medium. All cells were cultured in an incubator at 37 °C with 5% CO2.

Experimental animals were obtained from the Experimental Animal Center of Guangzhou University of Chinese Medicine (Guangzhou, China). All animal operations were approved by the institutional Animal Ethics Committee (Approval No. ZYD-2025-165) and conducted in full compliance with the ARRIVE Essential 10 guidelines.

### 2.3 Preparation of ACELNs, B-ACELNs and DIO/DIR labeled ACELNs

ACELNs were isolated from *Astragali Radix* and *Curcumae Rhizoma* using a modified differential centrifugation and sucrose density gradient method. Fresh *Curcumae Rhizoma* was homogenized to obtain juice, while dried *Astragali Radix* was soaked, decocted, and concentrated. Both samples were separately centrifuged at 500×g, 1000×g, and 5000×g for 10 min, 10 min, and 30 min at 4 °C to remove residues and impurities. The pre-purified solutions were mixed at a mass ratio of 2:1 and decocted under reduced pressure at 70 °C for 20 min. Crude ACELNs were collected by ultracentrifugation at 100,000×g for 60 min at 4 °C, then further purified using a continuous sucrose gradient (8%, 30%, 45%, 60%, w/v). The 30-45% sucrose fraction was collected, washed with PBS, and ultracentrifuged again. The final ACELNs were resuspended in cold PBS, lyophilized, and stored at −20 °C for further use. CELNs were prepared using the same procedure without the addition of *Astragali Radix* extract.

ACELNs was added to methanol, vortexed and sonicated at 75% power for 1 min (10 s on, 5 s off), and dried under vacuum at 70 °C. The solid powder obtained was the Broken *Astragali Radix-Curcumae Rhizoma* Exosome-like Nanoparticles (B-ACELNs).

ACELNs was dispersed in PBS, mixed with DIO/DIR (100 μg/mL) and incubated at 37 °C for 30 min in the dark. The samples were transferred to a 100 kDa ultrafiltration tube, centrifuged at 4 °C and 3,000 rpm for 10 min, the unconnected fluorescent dye were removed.

### 2.4 Characterization of ACELNs and CELNs

The physicochemical properties of ACELNs and CELNs were systematically characterized. Particle size and zeta potential were determined by dynamic light scattering (DLS) using a Zetasizer Nano ZS (Malvern Instruments, UK). Morphology was observed by transmission electron microscopy (TEM, Hitachi, Japan) after staining with 3% uranyl acetate. Total lipids were extracted with methanol/methyl tert-butyl ether (1:2, v/v), dried under nitrogen, and analyzed by thin-layer chromatography (TLC) on silica gel plates developed with chloroform/methanol/glacial acetic acid (95:4.5:0.5, v/v/v). Lipids were visualized by charring at 120 °C after spraying with 10% CuSO₄ in 8% phosphoric acid. Total protein extracts were separated by 12% SDS-PAGE and stained with Coomassie Brilliant Blue R-250. Total RNA was isolated using Trizol reagent. RNA concentration was quantified with a NanoDrop ND-2000 and further examined by agarose gel electrophoresis. For UPLC-MS/MS analysis (Thermo Fisher Scientific, USA), samples were extracted with methanol using ginsenoside Rb1 as an internal standard, sonicated, and centrifuged prior to injection.

### 2.5 Stability studies of ACELNs

Using phosphate buffer solution (PBS), simulated gastric fluid (SGF) and simulated intestinal fluid (SIF) as media, the same amount of ACELNs were evenly dispersed in each media. Incubation at 37 °C, 200 μL were taken from 0 h, 0.5 h, 1 h, 3 h, 6 h and 12 h respectively, the particle sizes of ACELNs in three media were mesured by DLS, and the morphology of ACELNs in 12 h media were observed by TEM. In addition, the particle size of ACELNs in PBS with pH of 1.2, 4.5, 6.8 and 7.4 were measured.

### 2.6 In vitro drug release of ACELNs

ACELNs and B-ACELNs were suspended in PBS (pH 7.4), simulated gastric fluid (SGF, pH 1.47), and simulated intestinal fluid (SIF, pH 6.48) containing 0.1% Tween 80 at 37 °C, then transferred into dialysis bags (MWCO 10 kDa). Dialysis was performed in 30 mL of corresponding medium at 37 °C. At set time intervals, samples were withdrawn and replaced with equal volumes of fresh medium. Samples were extracted with ethyl acetate, vacuum-dried, reconstituted in methanol, and analyzed by UPLC-MS/MS. The cumulative release percentage was calculated as (released amount at each time point / total initial amount) × 100%.

### 2.7 In vitro anti-tumour effects of ACELNs

Lo2, A549, HepG2, and Caco-2 cells were seeded in 96-well plates (8 × 103 cells/well) and treated with various concentrations of ACELNs or B-ACELNs for 24 h. Cell viability was measured by CCK-8 assay at 450 nm, with viability calculated as:

$$\left[\left(\text{ODtreated}-\text{ODblank}\right)/\left(\text{ODcontrol}-\text{ODblank}\right)\right]\times 100\%.\text{Viability was also determined}\;\mathrm{at}\;\text{multiple time points}\;\mathrm{at}\;1\;\mathrm{mg}/\mathrm{mL}$$

For scratch migration assays, A549 and HepG2 cells were seeded in 6-well plates (6 × 105cells/well) and treated with 0.1 mg/mL ACELNs or B-ACELNs. Migration was imaged at 0, 12, and 24 h, and migration rates were quantified using Image J.

For apoptosis and cell cycle analysis, A549 cells and HepG2 cells were inoculated into 6-well plates (4 × 105 cells/well) and treated with 0.1 mg/mL ACELNs or B-ACELNs for 24 h. Cells were harvested, stained with Annexin V-FITC/PI, and analyzed using a Dxp Athena flow cytometer (Shanghai Xiatai Biotechnology Co., Ltd., China) according to the manufacturer’s protocol.

### 2.8 Cellular uptake assessment

HepG2 and Lo2 cells were seeded onto coverslips in 24-well plates (5 × 104 cells/well) and incubated with DiO-labeled ACELNs (0.1 mg/mL) for 1, 4, 6, and 8 h. Cells were washed with cold PBS, fixed in 4% paraformaldehyde for 15 min, and washed with PBS, and mounted with DAPI-containing anti-fade mounting medium. Intracellular fluorescence was observed using a fluorescence microscope (Olympus, Japan).

HepG2 cells were seeded in 12-well plates (2 × 105 cells/well) and treated with ACELNs or B-ACELNs (0.1 mg/mL) for 1, 2, 4, 6, 8, and 12 h. Cells were harvested, lysed by sonication, extracted with ethyl acetate, and dried under vacuum. Intracellular levels of formononetin and calycosin were quantified by UPLC-MS/MS.

HepG2 cells and Lo2 cells were inoculated in a 12-well plates (3 × 105 cells/well) and pretreated with endocytosis inhibitors for 1 h (Sodium propionate 14.8 mg/mL, chlorpromazine hydrochloride 10 μg/mL, indomethacin 37.58 μg/mL (0.1% DMSO, v/v) and colchicine 360 μg/mL, DMEM medium without FBS). ACELNs and B-ACELNs (0.1 mg/mL, DMEM medium without FBS) were added and incubated for 6 h at 37 °C. The contents of formononetin and calycosin in the sample were determined according to the previous treatment.

### 2.9 Transport of ACELNs in Caco-2 Cell Monolayer Model

Caco-2 cells (1 × 105 cells/mL, 0.5 mL) were seeded onto the apical (AP) side of 12-well Transwell plates. The integrity of the cell monolayer was verified by measuring transepithelial electrical resistance (TEER) using a Millicell ERS-2 ohmmeter (Millipore, USA). After monolayer maturation, cells were rinsed and equilibrated with pre-warmed HBSS at 37 °C for 30 min. ACELNs or B-ACELNs (0.15 mg/mL in HBSS, 0.5 mL) were added to the AP side, and 1.5 mL blank HBSS to the basolateral (BL) side. Samples (200 μL) were taken from the BL side at 1, 2, 4, and 8 h, with equal fresh HBSS replenished immediately. The concentrations of formononetin and calycosin were quantified by UPLC-MS/MS. The apparent permeability coefficient (*P*_app_) was calculated as:

$$P_{\mathrm{app}}=\left(\text{Transport amount}\;\mathrm{per}\;\text{unit time}\right)/\left(\text{Initial concentration}\times \text{Membrane area}\right)$$

### 2.10 In vivo pharmacokinetic studies

Ten male Sprague–Dawley (SD) rats weighing 220 ± 20 g were randomly divided into two groups (n = 5) and fasted for 12 h with free access to water prior to the experiment. Rats were orally administered ACELNs or B-ACELNs at a dose of 1 g/kg. Blood samples (0.5 mL) were collected from the retro-orbital venous plexus into heparinized tubes at 5, 15, 30 min, 1, 2, 3, 4, 6, 8, 10, and 12 h post-administration. Samples were kept at 4 °C and centrifuged at 5000 rpm for 10 min. The obtained plasma was stored at −80 °C until analysis.

Plasma (200 μL) was mixed with 4 μL of internal standard ginsenoside Rb1 (2 μg/mL) and 800 μL methanol-acetonitrile (1:1, v/v). The mixture was vortexed for 1 min, sonicated for 10 min, and centrifuged at 12,000 rpm for 10 min. The supernatant was evaporated to dryness at 60 °C using a vacuum concentrator. The residue was reconstituted in 100 μL methanol, centrifuged at 12,000 rpm for 5 min, and 3 μL of the supernatant was analyzed by UPLC-MS/MS.

### 2.11 In Vivo Distribution of DiR-Labeled ACELNs

Twenty-four female C57BL/6 mice (20 ± 2 g) were randomly divided into two groups and fasted for 12 h with free access to water prior to the experiment. Mice were orally administered DiR-labeled ACELNs or free DiR at a DiR dose of 0.15 mg/kg. Mice were sacrificed at 3, 6, 12, and 24 h post-administration. The gastrointestinal tract and major organs (heart, liver, spleen, lung, kidney) were collected and subjected to ex vivo fluorescence imaging using an IVIS Spectrum system (n = 3).

### 2.12 In vivo antitumor activity of ACELNs

Lewis lung cancer xenografts were established in 6-week-old male C57BL/6J mice by subcutaneous inoculation of 1 × 10^6^ cells in the right axillary region. Thirty tumor-bearing mice were randomly divided into 5 groups (n = 6): model group (daily i.g. PBS + i.p. PBS on days 2 and 11), 5-FU group (daily i.g. PBS + i.p. 5-FU at 75 mg/kg on days 2 and 11), B-ACELNs group (daily i.g. 400 mg/kg B-ACELNs), ACELNs group (daily i.g. 400 mg/kg ACELNs), and ACELNs + 5-FU group (daily i.g. 400 mg/kg ACELNs + i.p. 5-FU on days 2 and 11). Treatment was started 2 days after inoculation and continued for 18 days under animal welfare guidelines to maintain tumor volume < 2000 mm^3^.

Body weight and tumor volume were recorded regularly. Tumor volume was calculated as (length × width^2^)/2. At the end of the experiment, blood was collected by cardiac puncture, and tumors, heart, liver, spleen, lung, and kidney tissues were harvested. Tumor weight was measured, and organ indices were calculated as (organ weight/body weight) × 100%. Histopathological changes in tumor tissues were examined by H&E staining, and in situ apoptosis was determined by TUNEL assay according to the manufacturer’s instructions.

### 2.13 In vivo safety evaluation

Serum levels of liver function markers (ALT, AST), renal function markers (UREA, CREA), and cardiac function markers (CK, LDH) were determined using commercial kits according to the manufacturer’s instructions. Complete blood count (CBC) was analyzed using an automated hematology analyzer. Harvested heart, liver, spleen, lung, and kidney tissues were fixed in 4% paraformaldehyde, dehydrated, paraffin-embedded, sectioned at 4-5 μm, and H&E-stained for histopathological examination.

### 2.14 In vivo antitumor mechanism studies

Cytokine levels including TNF-α, IFN-γ and TGF-β1 in tumor tissues and serum were determined using commercial ELISA kits. Tumor tissues were homogenized in PBS with protease inhibitors and centrifuged to obtain supernatants, while serum was separated from whole blood. Optical density was measured by a microplate reader, and cytokine concentrations were calculated using standard curves.

Immunohistochemical staining for CD31 and VEGF was performed on paraffin-embedded tumor sections. After antigen retrieval, antibody incubation and DAB development, microvessel density and VEGF expression were quantified in three random high-power fields per section.

Total RNA was extracted from tumor tissues using Trizol reagent, and RNA quality was verified by Nanodrop2000 and Agilent 5300 bioanalyzer. Qualified samples were used for library preparation and sequencing by Novogene on a NovaSeq X Plus platform. Gene expression was quantified by featureCounts, and DEGs were identified with DESeq2 (FDR < 0.05, |log₂FC| ≥ 1), followed by GO and KEGG enrichment analyses using clusterProfiler.

### 2.15 Data analyses

Statistical analysis was performed using IBM SPSS Statistics 23 software. All values were analyzed by one-way ANOVA or independent sample T test under the condition of normal distribution and homogeneous variance. The results were considered statistically significant when P < 0.05. All results are expressed as mean ± SD.

## 3 Results and discussion

### 3.1 Preparation and characterization of ACELNs

**Fig. 1:**
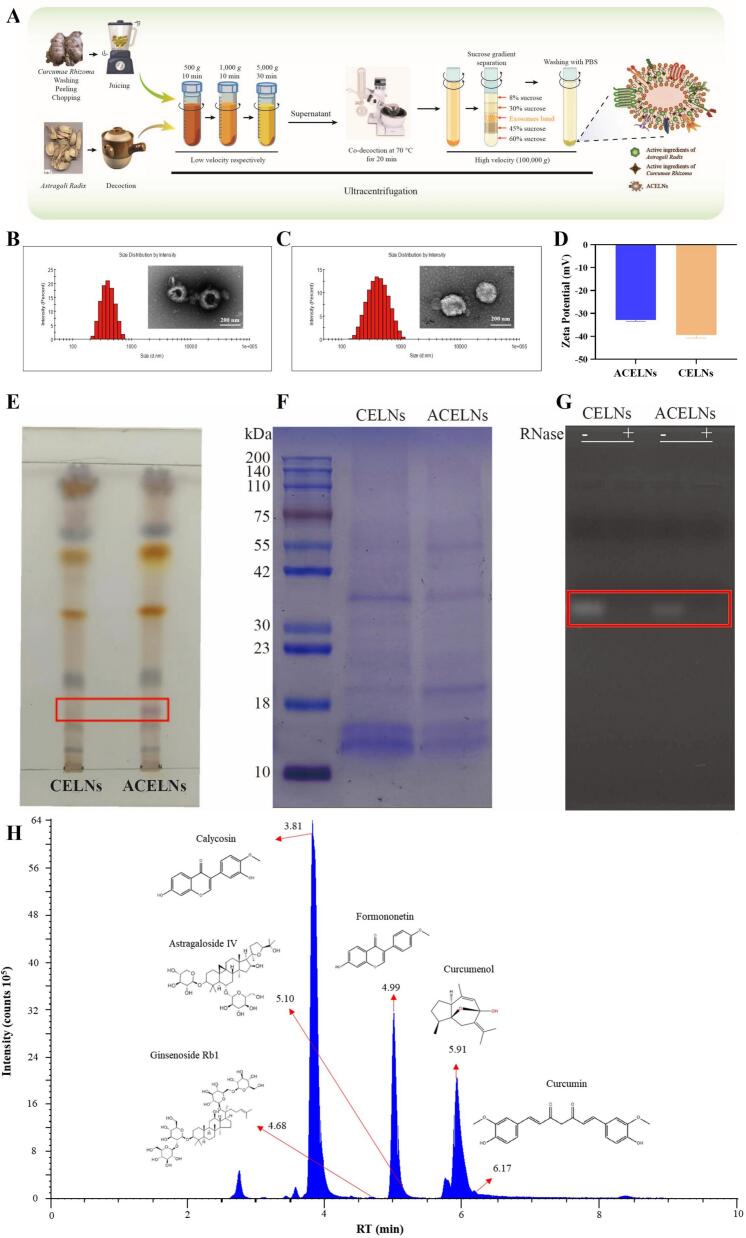
Characterization of ELNs. A. The extraction and separation process of ELNs. B-C. Particle size distribution and TEM observation of ACELNs (B) and CELNs (C). (Scale bar: 200 nm). D. The Zeta potential of ELNs. E. Lipids in ELNs were separated by TLC. F. Proteins in ELNs were separated by SDS-PAGE and detected by coomassie brilliant blue staining. G. RNA in ELNs were separated by Agarose gel Electrophoresis. H. The LC-MS spectrum of ACELNs.

ACELNs were successfully isolated from the co-decoction of fresh *Curcumae Rhizoma* and aqueous extract of *Astragali Radix* via differential ultracentrifugation and sucrose density gradient purification (Fig. 1A). Dynamic light scattering (DLS) analysis revealed that ACELNs and CELNs exhibited hydrodynamic diameters of 300-400 nm with PDI < 0.3, and negatively charged surfaces with absolute zeta potentials exceeding 30 mV, indicating excellent colloidal stability (Table S1, Fig. 1D). TEM observations showed that CELNs displayed a concave hemispherical or bowl-like morphology, whereas ACELNs presented a uniform flat spheroid structure (Fig. 1B, C). The distinct morphological discrepancy between single-herb vesicles and co-decoction-derived nanovesicles strongly implies that nanostructural rearrangement occurs during the co-decoction process, which is consistent with the previously reported self-assembly behavior of herbal nanoparticles induced by heating in aqueous decoction systems (Yu et al., 2026). The particle sizes observed by TEM (200-250 nm) were smaller than those determined by DLS, which could be attributed to morphological shrinkage during sample preparation and negative staining. Such typical spherical bilayer morphology and stable nanoscale particle size are the basic morphological hallmarks of plant-derived exosome-like nanoparticles, providing preliminary morphological evidence for the classification of ACELNs.

Thin-layer chromatography (TLC) demonstrated that ACELNs and CELNs shared identical lipid profiles, with ACELNs displaying one additional band (Fig. 1E). SDS-PAGE showed highly similar protein band patterns between ACELNs and CELNs, with major bands at 15, 17, 20, 38, and 55 kDa (Fig. 1F). Both nanoparticles contained intact RNA, as verified by agarose gel electrophoresis, and signals disappeared after RNase digestion (Fig. 1G). These results confirmed that the lipid, protein, and RNA components of ACELNs were predominantly derived from *Curcumae Rhizoma*. The complete lipid bilayer composition, characteristic vesicular protein bands, and encapsulated intact nucleic acids are core membrane biomarkers to distinguish exosome-like nanovesicles from simple herb precipitates or micelles, which greatly strengthens the identity basis of ACELNs as herbal ELNs.

UPLC-MS/MS verified the successful co-encapsulation of multiple bioactive components in ACELNs, including curcumenol and curcumin from *Curcumae Rhizoma*, and formononetin, calycosin, and astragaloside IV from *Astragali Radix* (Table 1). As shown in Table 1, distinct loading levels of the five characteristic phytochemicals were observed, which could be attributed to the differences in lipophilicity and molecular size of each compound. The lipophilic terpenoids and flavonoids were preferentially embedded into the lipid bilayer of ACELNs, while the high-molecular-weight astragaloside IV exhibited a relatively lower loading content. Combined with the subsequent in vitro release profiles, the intact vesicular structure can effectively retain these bioactive components and slow their leakage in gastrointestinal media, demonstrating the superior encapsulation and retention capacity of ACELNs. Further spectral characterizations, including FT-IR, UV-vis, fluorescence, and Raman spectroscopy, confirmed consistent chemical profiles between ACELNs and CELNs (Fig. S1A-E). Collectively, these data demonstrate that co-decoction-driven self-assembly enables ACELNs to efficiently encapsulate bioactive components from both herbs while retaining favorable colloidal properties.

**Table 1:** Content of active components of ELNs. Data are presented as mean ± SD (n = 3).

ELNs	Curcumenol (ng/mg)	Curcumin (ng/mg)	Formononetin (ng/mg)	Calycosin (ng/mg)	astragaloside IV (ng/mg)
ACELNs	3241.56± 56.84	695.70 ± 11.29	2148.74 ± 30.32	2957.75 ± 11.84	21.93 ± 2.94
CELNs	72082.54± 1847.43	35279.63 ± 4635.82	-	-	-

Taken together, the comprehensive characterizations of lipid constituents, membrane proteins, encapsulated nucleic acids, vesicle morphology and colloidal stability provide multi-dimensional evidence to solidly classify ACELNs as exosome-like nanoparticles originating from the co-decoction of *Astragali Radix* and *Curcumae Rhizoma*.

### 3.2 Stability and in vitro drug release of ACELNs

**Fig. 2:**
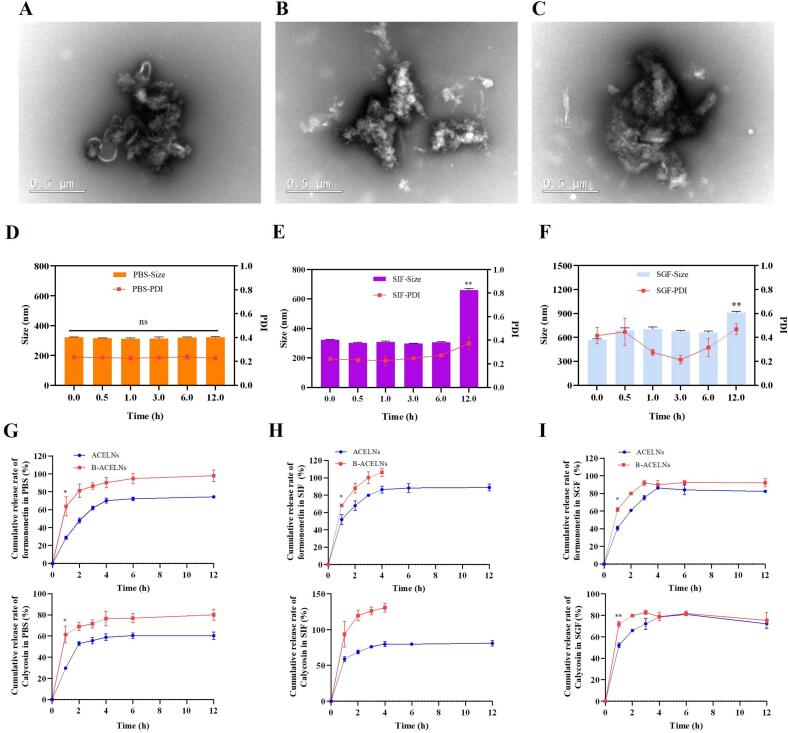
Colloidal stability and in vitro release behaviour of ACELNs. TEM images of ACELNs after 12 h incubation in PBS (A), SIF (B), and SGF (C) (scale bar: 0.5 μm). Time-dependent changes in particle size and polydispersity index (PDI) of ACELNs in PBS (D), SIF (E), and SGF (F). Cumulative release profiles of formononetin and calycosin from ACELNs and B-ACELNs in PBS (G), SIF (H), and SGF (I) over 12 h. Data are presented as mean ± SD (n = 3). (D-F) Compared with the previous time point, **P < 0.01 and ns represents no significant difference. (G-I) ACELNs compared with B-ACELNs at 1 h, *P < 0.05.

The colloidal stability of ACELNs was investigated in PBS, SGF and SIF (Fig. 2). ACELNs maintained a stable particle size in PBS for up to 12 h. In SGF and SIF, ACELNs remained stable within 6 h, but exhibited slight agglomeration and increased particle size at 12 h, which could be attributed to the acidic environment and enzymatic hydrolysis. Furthermore, the hydrodynamic diameter of ACELNs was pH-dependent and more variable under stronger acidic conditions (Fig. S2). These results confirm that ACELNs possess favourable gastrointestinal stability for oral delivery.

Quantitative statistical analysis of time-dependent particle size and PDI values further verified the above trend. Significant elevations in particle dimension and polydispersity index were only detected at the 12 h time point in SGF and SIF (*P* < 0.01), while no remarkable fluctuations were observed within the first 6 h of incubation, quantitatively demonstrating the acceptable anti-erosion capacity of ACELNs lipid bilayers in digestive fluids.

In vitro release profiles showed rapid initial release of formononetin and calycosin within 1 h, followed by sustained release up to 12 h in PBS, SGF, and SIF (Fig. 2). B-ACELNs displayed faster and higher release of bioactive components than intact ACELNs. To quantitatively clarify the underlying release mechanism, four classic kinetic models (zero-order, first-order, Higuchi, Korsmeyer-Peppas) were fitted to all release curves, and all fitting equations together with correlation coefficients (*r*^2^) are summarized in Supplementary Tables S3-S5. Fitting results demonstrated that intact ACELNs exhibited the highest correlation with the first-order model in all three simulated gastrointestinal media, and Korsmeyer-Peppas release exponent *n* values below 0.45 confirmed Fickian diffusion as the dominant release pathway. The intact nanostructure of ACELNs effectively slowed down the release of bioactive components in gastric and intestinal fluids, which is beneficial for targeted delivery and sustained retention in the gastrointestinal tract.

Combined with the quantitative colloidal stability data above, it can be concluded that the intact lipid vesicle structure simultaneously realizes structural stability in digestive environments and controlled sustained release of loaded phytochemicals, which is a critical advantage for improving the oral residence time and absorption efficiency of herbal active ingredients.

### 3.3 In vitro anti-tumour effects of ACELNs

**Fig. 3:**
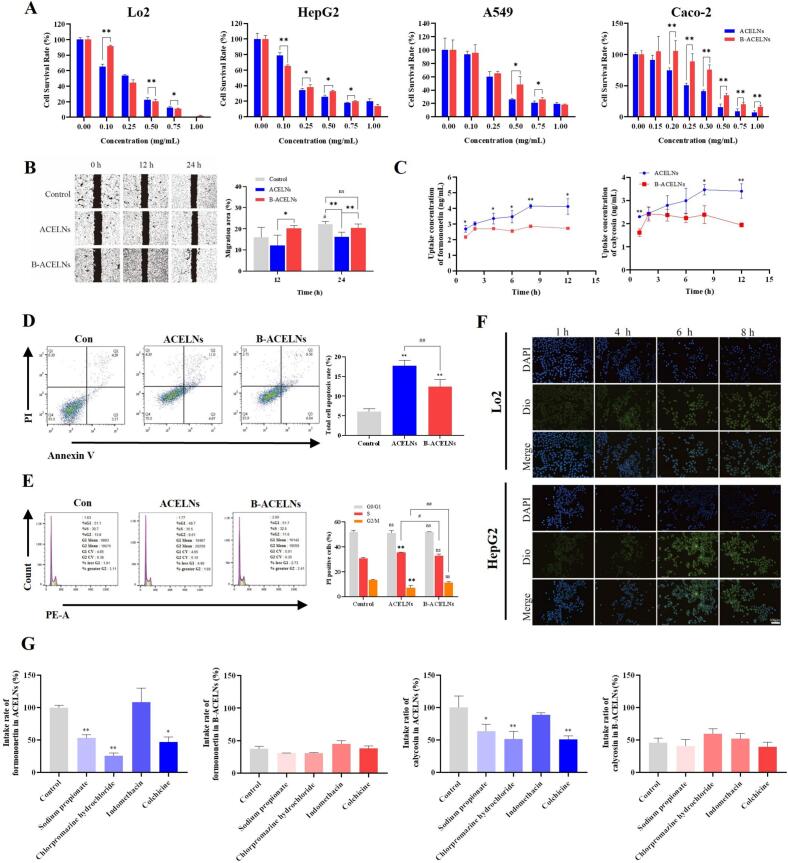
In vitro anti-tumour activity and cellular uptake of ACELNs. (A) Cell survival rates of normal (Lo2) and tumour (HepG2, A549, Caco-2) cells after treatment with ACELNs or B-ACELNs for 24 h. (B) Scratch wound healing assay showing migration inhibition of HepG2 cells by ACELNs and B-ACELNs at 12 h and 24 h. (C) Time-dependent intracellular uptake of formononetin and calycosin from ACELNs or B-ACELNs in HepG2 cells. (D, E) Flow cytometry analysis of apoptosis (D) and cell cycle distribution (E) in HepG2 cells after treatment with ACELNs or B-ACELNs. (F) Fluorescence imaging of DiO-labelled ACELNs internalization in Lo2 and HepG2 cells at 1, 4, 6, and 8 h. (G) Effects of endocytic inhibitors on the intracellular uptake of formononetin and calycosin from ACELNs or B-ACELNs in HepG2 cells. Data are presented as mean ± SD. For panels A and B, n = 6; *P < 0.05, **P < 0.01 (ACELNs vs. B-ACELNs), #*P* < 0.05 (vs. Control group). For panels C-G, n = 3; Compared between groups, compared with previous time or compared with control group. *P < 0.05, **P < 0.01, #*P* < 0.05, ##*P* < 0.01 and ns represents no significant difference.

The in vitro anti-tumour activity and structure-dependent bioactivity of ACELNs were evaluated in tumour cells (HepG2, A549, Caco-2) and normal Lo2 hepatocytes, with B-ACELNs as control (Fig. 3, Table S2). CCK-8 assays revealed that ACELNs exerted dose-dependent cytotoxicity towards HepG2, A549 and Caco-2 cells, with IC_50_ values of 0.1260, 0.2584 and 0.2335 mg/mL, respectively. In contrast, ACELNs showed significantly lower toxicity to normal Lo2 cells (IC_50_ = 0.5324 mg/mL), which was 3.23-fold higher than that in HepG2 cells. B-ACELNs exhibited reduced cytotoxicity against tumour cells but higher toxicity towards Lo2 cells relative to intact ACELNs. These results confirm that ACELNs exert favourable tumour-selective cytotoxicity dependent on structural integrity.

Scratch wound-healing assays showed that ACELNs significantly inhibited the migration of A549 and HepG2 cells (Fig. 3B, Fig. S4A, B). In A549 cells, ACELNs markedly reduced migration at 12 and 24 h relative to control and B-ACELNs groups (*P* < 0.05). In HepG2 cells, ACELNs induced stronger migration inhibition at 24 h compared with both control and B-ACELNs groups (*P* < 0.01), whereas B-ACELNs showed no significant effect. These results indicate that intact ACELNs effectively suppress tumour cell migration, which may contribute to reduced metastatic potential in vivo.

Flow cytometric analysis using Annexin V-FITC/PI staining revealed that ACELNs significantly promoted apoptosis in A549 and HepG2 cells (Fig. 3D, Fig. S3C, D). The total apoptosis rate of A549 cells reached 26.43 ± 0.16% after ACELNs treatment, markedly higher than control (8.29 ± 0.38%) and B-ACELNs groups (16.57 ± 0.34%) (*P* < 0.01). Similarly, the apoptosis rate in HepG2 cells increased to 18.10 ± 0.81%, compared with 6.11 ± 0.66% (control) and 12.45 ± 1.78% (B-ACELNs) (*P* < 0.01). Late apoptosis was the dominant population in both cell lines, indicating that ACELNs trigger progressive tumour cell apoptosis in a structure-dependent manner.

Cell cycle analysis showed that ACELNs induced distinct arrest profiles in a cell-specific manner (Fig. 3E, Fig. S3E, F). In A549 cells, ACELNs significantly increased G0/G1-phase distribution and reduced S and G2/M-phase populations (*P* < 0.01), with stronger effects than B-ACELNs. In HepG2 cells, ACELNs did not alter G0/G1 distribution but significantly increased S-phase cells and decreased G2/M-phase cells (*P* < 0.01). These findings demonstrate that ACELNs inhibit tumour cell proliferation via cell-specific cell cycle arrest, which is closely related to the intact nanostructure of ACELNs.

### 3.4 Cellular uptake assessment

Cellular uptake efficiency and internalization mechanisms of ACELNs were investigated in Lo2 and HepG2 cells using fluorescence imaging and UPLC-MS/MS quantification of formononetin and calycosin (Fig. 3C, F). Time-dependent fluorescence intensity analysis showed that intracellular uptake of ACELNs increased gradually in both cell lines within 8 h (Fig. S5). Uptake was comparable between Lo2 and HepG2 cells during the first 4 h, but became significantly higher in HepG2 cells at 6 h, indicating tumour-cell preferential internalization.

Quantitative analysis confirmed that uptake of bioactive components from B-ACELNs plateaued within 2 h, whereas uptake from intact ACELNs increased continuously and peaked at 8 h. The total intracellular accumulation of bioactive components was significantly higher in the ACELNs group than in the B-ACELNs group (*P* < 0.05), demonstrating that structural integrity of ACELNs greatly enhances cellular internalization.

Endocytosis inhibition studies revealed that uptake of bioactive components from ACELNs was significantly reduced by sodium propionate (energy-dependent endocytosis), chlorpromazine hydrochloride (clathrin-mediated endocytosis), and colchicine (microtubule-dependent endocytosis), but not by indomethacin (caveolae-mediated endocytosis) (Fig. 3G) (Fang et al., 2024). In contrast, uptake from B-ACELNs was unaffected by all inhibitors (*P* > 0.05). Similar uptake patterns were observed in Lo2 cells (Fig. S6). These results indicate that intact ACELNs are internalized mainly through energy-dependent clathrin and microtubule-mediated endocytosis, whereas bioactive components from B-ACELNs likely enter cells via passive diffusion. Overall, the intact nanostructure of ACELNs is essential for efficient cellular uptake of bioactive components from *Astragali Radix-Curcumae Rhizoma*. (Su et al., 2023)

The identification of clathrin-dependent and microtubule-associated energy-consuming internalization pathways reveals the core cellular-level mechanism underlying the superior intracellular delivery capacity of intact ACELNs, which acts as a prerequisite for their enhanced intestinal absorption efficiency in vivo.

### 3.5 Transport of ACELNs in Caco-2 Cell monolayer model

**Fig. 4:**
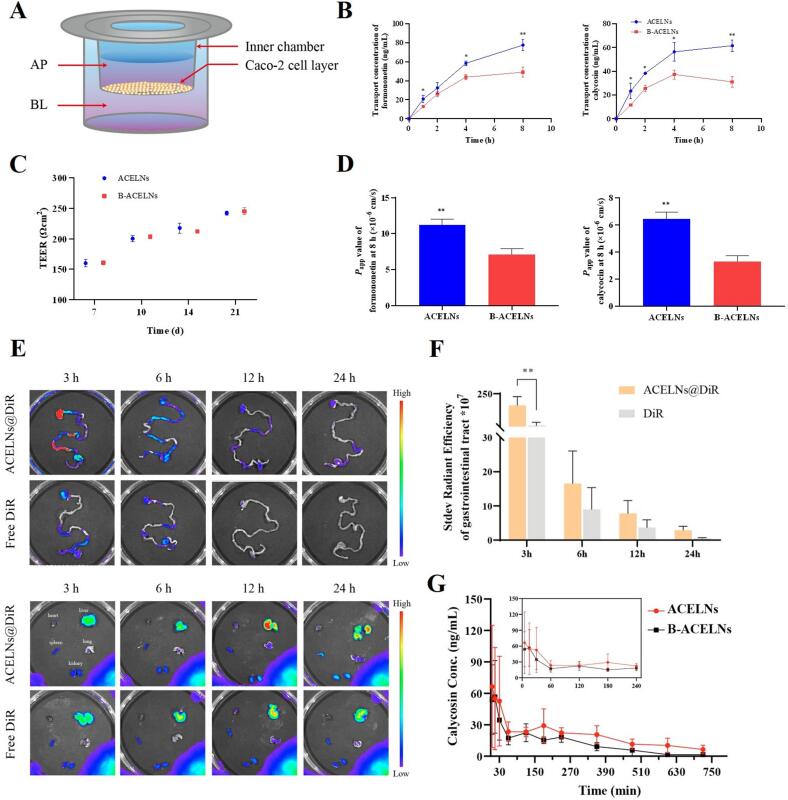
Intestinal transport, in vivo distribution, and pharmacokinetics of ACELNs. (A) Schematic diagram of the Caco-2 cell monolayer transwell model. (B) Time-dependent transport concentrations of formononetin and calycosin across Caco-2 monolayers. (C) TEER values of Caco-2 cell monolayers during culture, confirming barrier integrity. (D) Apparent permeability coefficients (*P*ₐₚₚ) of formononetin and calycosin at 8 h. (E) In vivo fluorescence imaging of gastrointestinal tract and major organs in C57BL/6 mice after administration of ACELNs@DiR or free DiR at 3, 6, 12, and 24 h. (F) Quantitative analysis of gastrointestinal radiant efficiency from the fluorescence images in (E). (G) Plasma concentration-time curve of calycosin in rats after intragastric administration of ACELNs or B-ACELNs. Data are presented as mean ± SD. For panels A-F, n = 3; *P < 0.05, **P < 0.01 (ACELNs vs. B-ACELNs or ACELNs@DiR vs. free DiR). For panel G, n = 5.

A Caco-2 cell monolayer model was employed to simulate the intestinal epithelial barrier and evaluate the transmembrane transport of ACELNs-loaded bioactive components (Fig. 4A, B) (Fang et al., 2024). Monolayer integrity was verified by transepithelial electrical resistance (TEER) measurements, which exceeded 240 Ω·cm^2^ on day 21 without significant intergroup differences (*P* > 0.05), confirming reliable barrier function for transport studies (Fig. 4C). Time-dependent transport and apparent permeability coefficients (*P*_app_) of formononetin and calycosin were quantified by UPLC-MS/MS (Fig. 4B, D). The B-ACELNs group reached maximal transport at 4 h, whereas the ACELNs group exhibited continuously increasing transport across the monolayer. The cumulative transport of bioactive components was significantly higher in the ACELNs group than in the B-ACELNs group (*P* < 0.01). At 8 h, the *P*_app_ values of formononetin and calycosin in the ACELNs group were (11.18 ± 0.86) × 10^-6^ cm/s and (6.44 ± 0.50) × 10^-6^ cm/s, respectively, which were markedly higher than those in the B-ACELNs group (*P* < 0.01). These results demonstrate that intact ACELNs significantly enhance the transmembrane transport of bioactive components across the intestinal epithelial barrier, and this promoting effect is highly dependent on the structural integrity of ACELNs.

Integrated with the ex vivo gastrointestinal fluorescence imaging data presented in Section 3.7, the amphipathic lipid bilayer of ACELNs confers favorable mucoadhesive properties, prolonging luminal residence time and extending the absorption window of encapsulated phytoconstituents. Collectively, synergistic effects including prolonged intestinal mucus retention, clathrin-mediated epithelial cell uptake, and elevated transepithelial permeability form a complete mechanistic cascade that accounts for the markedly improved oral bioavailability of herbal bioactive components delivered by ACELNs.

### 3.6 In vivo pharmacokinetic studies

In vivo pharmacokinetic studies were performed in rats to evaluate the effect of ACELNs on the oral absorption and bioavailability of calycosin, a representative bioactive component from *Astragali Radix*, following oral administration of ACELNs or B-ACELNs. Plasma concentrations were quantified by UPLC-MS/MS, and pharmacokinetic parameters were calculated using a non-compartmental model (Fig. 4G, Table 2). The plasma concentration-time profile of calycosin differed markedly between groups. The *C*_max_ of calycosin in the ACELNs group was slightly higher than in the B-ACELNs group, but the difference was not significant (*P* > 0.05). In contrast, *T*_max_ was significantly prolonged from 0.18 ± 0.09 h (B-ACELNs) to 0.78 ± 1.25 h (ACELNs), indicating delayed but sustained absorption. The *AUC*_(0–t)_ of calycosin in the ACELNs group was 234297.98 ± 68020.63 ng/L·h, which was 0.62-fold higher than in the B-ACELNs group (*P* < 0.05). The *MRT*_(0–∞)_ and *t*_1/2z_ were also significantly extended in the ACELNs group. The relative bioavailability (*Fr*) of calycosin in the ACELNs group reached 161.90%. Notably, only calycosin prototype could be stably quantified in rat plasma within the sampling period, other encapsulated components were undetectable, likely due to low plasma concentrations below instrument LOQ or rapid in vivo biotransformation into metabolites. These results confirm that intact ACELNs markedly improve the oral bioavailability of bioactive components by protecting them during gastrointestinal transit and enhancing intestinal absorption.

**Table 2:** Results of pharmacokinetic parameters of Calycosin in plasma after oral administration of ACELNs and B-ACELNs. Data are presented as mean ± SD (n = 5). ACELNs compared with B-ACELNs, *P < 0.05.

Parameters	B-ACELNs	ACELNs
*AUC*_(0-t)_ (ng/L*h)	144717.44 ± 28271.81	234297.98 ± 68020.63*
*AUC*_(0-∞)_ (ng/L*h)	150456.94 ± 31723.12	304974.11 ± 109449.89*
*MRT*_(0-∞)_ (h)	3.93 ± 1.78	8.40 ± 6.36
*t*_1/2z_ (h)	2.09 ± 1.31	5.60 ± 5.00
*T*_max_ (h)	0.18 ± 0.09	0.78 ± 1.25
*C*_max_ (ng/L)	60540.88 ± 37030.98	79285.83 ± 60156.31
*Fr* (%)	—	161.90

### 3.7 In vivo distribution of DIR labeled ACELNs

In vivo tissue distribution of orally administered ACELNs was investigated in C57BL/6 mice using DiR-labeled ACELNs (ACELNs@DiR) and free DiR, with fluorescence imaging at designated time points (Fig. 4E, F, Fig. S7). Fig. S7 provides quantitative radiant efficiency data for organ biodistribution. The gastrointestinal tract was the primary distribution site for ACELNs@DiR. Fluorescence intensity in the gastrointestinal tract was significantly stronger in the ACELNs@DiR group than in the free DiR group at all time points, especially at 3 h (*P* < 0.01). ACELNs@DiR exhibited prolonged retention in the gastrointestinal lumen, whereas free DiR was rapidly eliminated, providing an extended window for intestinal absorption of bioactive components. ACELNs@DiR mainly accumulated in the liver, indicating successful crossing of the intestinal barrier and entry into the systemic circulation via the portal vein. Notably, ACELNs@DiR showed preferential accumulation in the lung at 3 h (*P* < 0.05), suggesting early lung-targeting potential. At 24 h, fluorescence signals in the kidney were significantly higher in the ACELNs@DiR group (*P* < 0.05), indicating renal excretion as the main elimination pathway. No excessive fluorescence accumulation was observed in major organs, confirming that ACELNs are primarily retained in the gastrointestinal tract with limited systemic distribution. This favorable pattern enhances the safety of ACELNs and supports targeted delivery of bioactive components without obvious off-target effects.

### 3.8 In vivo antitumor activity of ACELNs

**Fig. 5:**
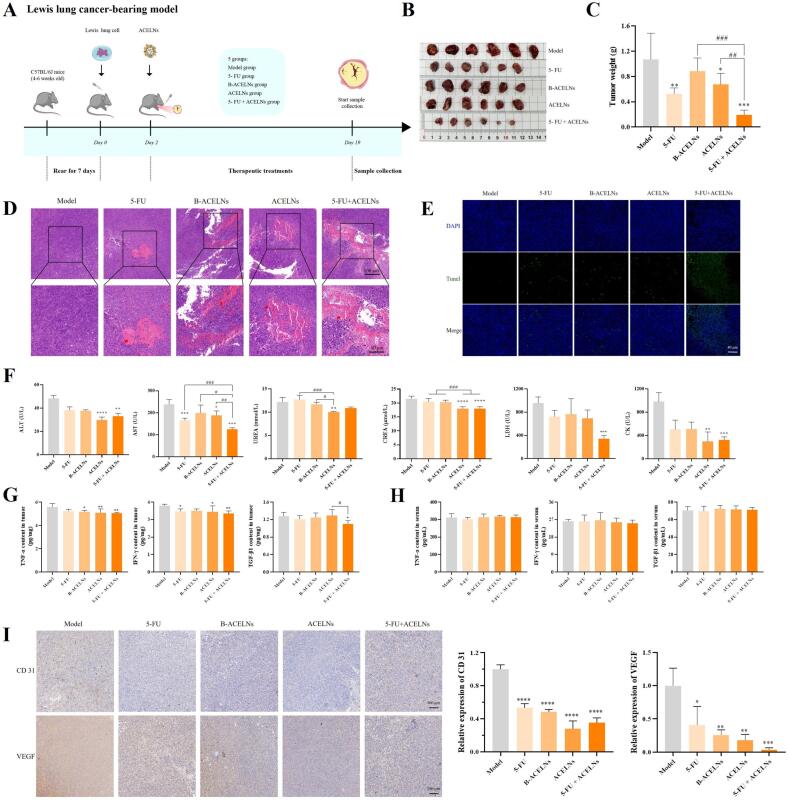
Synergistic antitumour efficacy and underlying mechanism of ACELNs combined with 5-FU in Lewis lung cancer mice. (A) Schematic illustration of the experimental design: C57BL/6 mice were subcutaneously inoculated with Lewis lung cancer cells, acclimatized for 7 days, and randomly divided into five groups (Model, 5-FU, B-ACELNs, ACELNs, 5-FU + ACELNs) for treatment. Samples were collected on day 19. (B) Representative images of excised tumours from each group. (C) Statistical analysis of tumour weight. (D) H&E staining of tumour tissues (scale bars: 100 μm, 50 μm). (E) TUNEL assay for tumour cell apoptosis (DAPI: nuclear staining; TUNEL: apoptotic cells; scale bar: 40 μm). (F) Serum biochemical analysis: ALT/AST (liver function), UREA/CREA (kidney function), and CK/LDH (cardiac/muscle function). (G, H) ELISA analysis of TNF-α, IFN-γ, and TGF-β1 levels in tumour tissues (G) and serum (H). (I) Immunohistochemical staining and quantitative analysis of CD31 and VEGF expression in tumour tissues (scale bar: 100 μm). Data are presented as mean ± SD. For panels A-H, n = 6; for panel I, n = 3. *P < 0.05, **P < 0.01, ***P < 0.001, ****P < 0.0001 vs. Model group; #*P* < 0.05, ##*P* < 0.01, ###*P* < 0.001 vs. between indicated groups.

The in vivo antitumour efficacy of ACELNs alone and in combination with 5-fluorouracil (5-FU) was evaluated in Lewis lung carcinoma-bearing C57BL/6 mice (Fig. 5A). Tumour volume and weight were measured to assess therapeutic effects (Fig. 5B, C, Fig. S8). The 5-FU + ACELNs combination group showed the strongest tumour suppression, with final tumour weight of 0.19 ± 0.07 g and an inhibition rate of 81.97% (*P* < 0.001 vs. model group). ACELNs monotherapy inhibited tumour growth by 36.9% (*P* < 0.05), whereas B-ACELNs exerted no significant effect (17.25%, *P* > 0.05). The combination group exhibited superior efficacy to 5-FU monotherapy (51.06%), confirming synergistic antitumour activity between ACELNs and 5-FU.

Mice treated with ACELNs maintained stable body weight, while 5-FU alone induced mild weight loss, indicating favourable biocompatibility of ACELNs. Histopathological and TUNEL analyses further supported these results (Fig. 5D, E). Tumours from the combination group showed extensive necrosis and reduced cell density, in sharp contrast to the intact, densely packed structure observed in the model group.

We calculated the theoretical additive tumour inhibition rate based on the Bliss Independence model, defined by the formula: Inh₁ + Inh₂ − Inh₁ × Inh₂. The theoretical inhibitory rate calculated from single-agent groups was 69.12% (36.9% for ACELNs monotherapy, 51.06% for 5-FU monotherapy), which was lower than the actual tumour inhibition rate of the combination group (81.97%). This result formally verifies that ACELNs combined with 5-FU exert genuine synergistic anti-tumour activity rather than a simple additive effect. Such synergistic efficacy stems from multiple complementary biological functions of ACELNs that amplify the tumoricidal potency of 5-FU. Notably, B-ACELNs carrying identical herbal phytoconstituents but disrupted nanovesicle structure displayed markedly impaired intestinal absorption and anti-tumour capacity, proving that intact ACELN nanoparticles exert unique delivery advantages independent of the intrinsic bioactivity of encapsulated herbal components. We further conducted systematic mechanistic investigations covering tumour cell apoptosis, proliferation, angiogenesis and intratumoural immune remodelling to dissect the underlying regulatory pathways in depth.

### 3.9 In vivo safety evaluation

Systematic safety assessments were performed in tumour-bearing mice, including serum biochemistry, hematology, and histopathological examination of major organs (Fig. 5F, Fig. S9-S11). Serum biochemical analysis showed that ALT and AST levels were significantly lower in all treatment groups than in the model group, with the 5-FU + ACELNs combination yielding the greatest improvement in liver function. CREA (renal marker) was reduced in the ACELNs and 5-FU + ACELNs groups, and UREA was decreased in the ACELNs group. LDH and CK levels were markedly lower in the 5-FU + ACELNs group, indicating alleviated cardiac and muscle injury. The reduced LDH also reflected suppressed tumour metabolism and necrosis (Nesakumar et al., 2014). Overall, the 5-FU + ACELNs combination most effectively improved organ function while exerting antitumour activity, with favourable biosafety. Hematological analysis revealed that 5-FU monotherapy caused significant myelosuppression, as shown by reduced WBC, RBC, HGB, and PLT counts. In contrast, ACELNs alone did not induce myelosuppression, and the 5-FU + ACELNs combination restored blood cell counts to normal levels, demonstrating that ACELNs can reverse 5-FU-induced immunosuppression. H&E showed no obvious lesions in the heart, liver, spleen, lung, or kidney after ACELNs or B-ACELNs treatment. The 5-FU group exhibited mild interstitial changes in the heart, alveolar septal thickening in the lung, and reduced splenic cellularity. These abnormalities were markedly attenuated in the 5-FU + ACELNs group. Collectively, these results confirm the high short-term biocompatibility of ACELNs and their ability to mitigate the systemic toxicity of 5-FU within the limited experimental cycle. Notably, the current safety evaluation only covers short-term detection indicators, and more comprehensive toxicological tests are still required to fully validate the long-term biosafety and clinical translation potential of ACELNs. Further systematic studies including repeated-dose toxicity, chronic toxicity, immunogenicity assessment, and long-term biodistribution monitoring will be carried out in our follow-up work to supply sufficient preclinical safety evidence for ACELNs.

### 3.10 In vivo antitumor mechanism studies

**Fig. 6:**
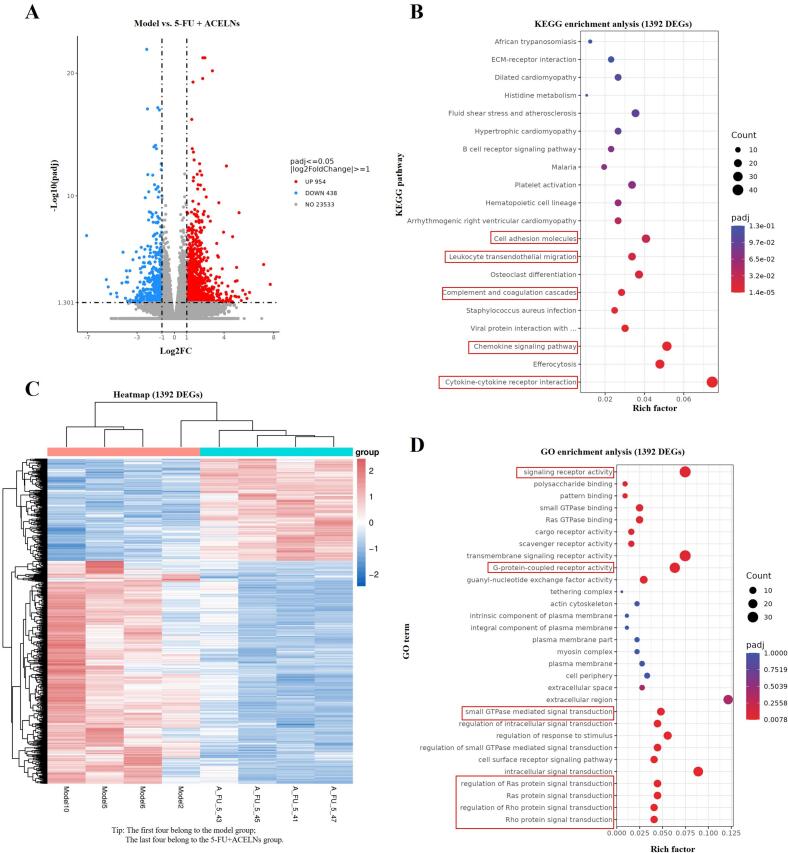
Transcriptomic analysis of combined treatment with ACELNs and 5-FU. (A) Volcano plot showing 1392 differentially expressed genes (DEGs) (filtering criteria: padj < 0.05, |log₂FC| ≥ 1). (B) KEGG pathway enrichment of DEGs, primarily enriched in cell adhesion molecules, leukocyte transendothelial migration, and cytokine-cytokine receptor interaction. (C) Heatmap displaying the distinct clustering of DEG expression profiles between Model (left 4) and 5-FU + ACELNs (right 4) groups. (D) GO enrichment analysis showing enrichment of terms related to signaling receptor activity and small GTPase-mediated signal transduction.

Immunohistochemical staining revealed high expression of CD31 (microvessel marker) and VEGF (angiogenic factor) in tumour tissues from the model group (Fig. 5I). All treatments downregulated CD31 and VEGF levels, with the strongest inhibition observed in the 5-FU + ACELNs group. ACELNs alone significantly reduced microvessel density, while the combination almost completely suppressed VEGF expression (3.33% of model), indicating potent anti-angiogenic activity. ELISA analysis showed that tumour tissue levels of TNF-α and IFN-γ were highest in the model group, reflecting an activated but ineffective tumour immune microenvironment (Fig. 5G). ACELNs and 5-FU + ACELNs treatments significantly reduced these cytokines, consistent with effective tumour killing. TGF-β1 (immunosuppressive cytokine) was also markedly decreased in the combination group. Serum cytokine levels remained unchanged among all groups (Fig. 5H), indicating no systemic immune disturbance. Transcriptome sequencing was performed to further explore the synergistic mechanism between ACELNs and 5-FU (Fig. 6). A total of 1392 differentially expressed genes (DEGs) were identified between the model and combination groups. KEGG analysis showed enrichment in cytokine-cytokine receptor interaction, leukocyte transendothelial migration, and chemokine signalling pathways. GO terms were mainly associated with signalling receptor activity and small GTPase-mediated signal transduction, suggesting that ACELNs could exert anti-tumour effects by regulating the immune microenvironment and cell signal transduction (Fu et al., 2025; Orlen et al., 2025; Parise et al., 2025; Yang and Wu, 2024). Similar to macrophage-derived EVs mediating pulmonary innate immune crosstalk as reported previously, ACELNs regulate multiple immune-related signaling cascades in lung tumor tissue to balance local inflammatory response (Lv et al., 2025). These results demonstrate that ACELNs synergize with 5-FU to inhibit tumour growth by suppressing angiogenesis (Luo et al., 2024) and remodelling the local immune microenvironment, without inducing systemic immune disorders. Notably, the RNA-seq data here are preliminary screening results, and further validation via qRT-PCR or Western blot will be conducted in follow-up work.

## 4 Conclusions

In summary, ACELNs were successfully isolated and characterized from the classic herbal pair of *Astragali Radix-Curcumae Rhizoma*. Notably, these plant-derived nanovesicles are formed through a self-reassembly process during co-decoction, which enables efficient loading of bioactive components from both herbs. ACELNs exhibited uniform nanostructure, favorable colloidal stability, and efficient cellular internalization via multiple endocytic pathways. Orally administered ACELNs significantly improved the oral bioavailability of active constituents, with enhanced gastrointestinal absorption and favorable tissue distribution in vivo. In vitro and in vivo evaluations demonstrated that ACELNs exerted selective anti-lung cancer effects with excellent biosafety, and synergistically enhanced the antitumor activity of 5-FU by regulating tumor microenvironmental cytokines (TNF-α, IFN-γ, TGF-β1) and inhibiting tumor angiogenesis (CD31, VEGF). Transcriptomic analysis further revealed the molecular pathways underlying the therapeutic effects of ACELNs. This work bridges TCM compatibility theory with modern nanomedicine, providing a novel phytogenic nanovesicle platform that efficiently improves the oral bioavailability of herbal active components.

## CRediT authorship contribution statement

**Ruiyue Fang:** Writing – original draft, Validation, Formal analysis, Data curation, Conceptualization. **Jingbei Zhang:** Writing – review & editing, Project administration. **Yiyang Cen:** Resources, Investigation, Formal analysis. **Xufeng Yang:** Writing – original draft, Validation. **Xiaoping Fang:** Writing – original draft, Validation. **Ziyang Wu:** Validation, Formal analysis, Data curation. **Chunyu Huang:** Visualization, Supervision. **Junfeng Wu:** Visualization, Supervision. **Yuhua Yang:** Visualization, Supervision. **Zhuowei Li:** Visualization, Supervision. **Long Xi:** Writing – review & editing. **Yan Ma:** Writing – review & editing, Project administration. **Shixia Guan:** Writing – review & editing, Project administration, Funding acquisition. **Liping Cao:** Writing – review & editing, Project administration.

## Ethical approval statement

The animal experiment scheme and process were approved by the Experimental Animal Center of Guangzhou University of Chinese Medicine and the guiding principles of the Animal Ethics Committee.

## Funding statement

The work was supported by the 10.13039/501100001809National Natural Science Foundation of China (No.82173986).

## Declaration of competing interest

The authors declare no competing financial interests or personal relationships that could influence the work reported in this paper.

## Data Availability

Data will be made available on request. Raw data supporting the conclusions are available from the corresponding author upon reasonable request.
