# Supplementary material for: Exosome-like nanoparticles derived from Astragali Radix-Curcumae Rhizoma co-decoction enhance oral bioavailability and provide synergistic efficacy and reduced toxicity in combination with 5-fluorouracil for lung cancer therapy
Source: Int J Pharm X. 2026 Jul 13;12:100611. doi: 10.1016/j.ijpx.2026.100611 (PMC13393709; doi:10.1016/j.ijpx.2026.100611)
Supplement: Supplementary file 1 — Supplementary material [file mmc1.docx]

**Exosome-like nanoparticles derived from *Astragali Radix-Curcumae Rhizoma* co-decoction enhance oral bioavailability and provide synergistic efficacy and reduced toxicity in combination with 5‑fluorouracil for lung cancer therapy**

Ruiyue Fang ^a, c^, Jingbei Zhang ^b^, Yiyang Cen ^a^, Xufeng Yang ^a^, Xiaoping Fang ^a^, Ziyang Wu ^a^, Chunyu Huang ^a^, Junfeng Wu ^a^, Yuhua Yang ^a^, Zhuowei Li ^a^, Long Xi ^a, *^, Yan Ma ^a, *^, Shixia Guan ^a, *^, Liping Cao ^c, *^

^a^ *School of Pharmaceutical Sciences, Guangzhou University of Chinese Medicine, Guangzhou 510006, China*

^b^ *The Second Affiliated Hospital of Guangzhou University of Chinese Medicine (The Second Clinical Medical College, Guangzhou University of Chinese Medicine), Guangzhou* *510120, China*

^c^ *Shenzhen Bao'an Traditional Chinese Medicine Hospital, Guangzhou University of Chinese Medicine, Shenzhen, Guangdong 518000, China*

^*^ Corresponding authors.

*E-mail addresses:* [xilong@gzucm.edu.cn](mailto:xilong@gzucm.edu.cn) (L. Xi), [mayan2006@gzucm.edu.cn](mailto:mayan2006@gzucm.edu.cn) (Y. Ma), [drguan@gzucm.edu.cn](mailto:drguan@gzucm.edu.cn) (S. Guan), [caoliping86@gzucm.edu.cn](mailto:caoliping86@gzucm.edu.cn) (L. Cao)

**Table of contents**

[**1. Experimental materials and methods** 3](#_Toc233301872)

[**2. Supporting tables and figures** 6](#_Toc233301873)

[**3. Abbreviations** 13](#_Toc233301874)

[**4. List of core chemical compounds** 14](#_Toc233301875)

# **1. Experimental materials and methods**

1.1. Materials

Crude Drug of *Curcumae Rhizoma* (“Ezhu” in Chinese) was from Cenxi City, Guangxi (China). *Astragali Radix* (*Astragalus Membranaceus* Decoction Pieces, Lot. 230103, “Huangqi” in Chinese) were from Gansu province (China). Phosphate Buffered Saline (PBS) was obtained from Thermo Fisher Scientific Co., Ltd (Shanghai, China). Sucrose, Colchicine were purchased from Macklin Biochemical Technology Co., Ltd (Shanghai, China). Trichloromethane (Chloroform), Methanol, Isopropanol, Tween 80, Ethyl Acetate Copper Sulfate, Phosphoric Acid were acquired from Guangzhou Chemical Reagent Factory (Guangzhou, China). Glacial Acetic Acid was obtained from Tianjin Damao Chemical Reagent Factory (Tianjin, China). Methyl Tert-Butyl Ether, KBr (Spectral Grade), Quercetin were purchased from Shanghai Aladdin Biochemical Technology Co., Ltd (Shanghai, China). Sterile Enzyme-Free Water, 12% One-Step Gel Preparation Kit were obtained from Hangzhou Fude Biotechnology Co., Ltd (Hangzhou, China). Agarose was from Beijing Yami Biotechnology Co., Ltd (Beijing, China). 10×TBE Buffer Premixed Powder, Nucleic Acid GelRed Dye, Trizol Reagent, Ribonuclease A were purchased from Sangon Biotech Co., Ltd (Shanghai, China). Protease Inhibitor, 0.25% Trypsin-NO EDTA, Annexin V-FITC/PI Cell Apoptosis Detection Kit, Cell Cycle and Apoptosis Detection Kit, Calycosin Reference Substance, Formononetin Reference Substance, Astragaloside IV Reference Substance were obtained from Dalian Meilun Biotechnology Co., Ltd (Dalian, China). RIPA Lysis Buffer, 5×Protein Loading Buffer, Bovine Serum Albumin were purchased from Shanghai Biyuntian Biotechnology Co., Ltd (Shanghai, China). BCA Protein Concentration Assay Kit, DMSO were from Sigma (USA) and Sigma-Aldrich (USA), respectively. Protein Marker was from Thermo (USA). 5×RNALoading Buffer was from Anhui Baike Biotechnology Co., Ltd (China). Tetramethylethylenediamine (TEMED), Ammonium Persulfate (APS) were purchased from Shanghai Aladdin Biochemical Technology Co., Ltd (Shanghai, China). Coomassie Brilliant Blue R250 was from Hangzhou Biodo Biotechnology Co., Ltd (Hangzhou, China). Silica Gel G Plate was obtained from Qingdao Ocean Chemical Co., Ltd (Qingdao, China). Chromatographic Methanol, Chromatographic Acetonitrile were from Merck (Germany). Chromatographic Formic Acid was obtained from Ron Chemical Co., Limited (Shanghai, China). Curdione Reference Substance, Curcumol Reference Substance, Curcumin Reference Substance, Curcumol Reference Substance, Germacrone Reference Substance were purchased from Chengdu Push Bio-Technology Co., Ltd (Chengdu, China). Ginsenoside Rb1 Reference Substance was from National Institutes for Food and Drug Control (China). Fetal Bovine Serum (FBS) was obtained from Nanjing Shenghang Biotechnology Co., Ltd (Nanjing, China). 0.25% Trypsin-EDTA, Penicillin/Streptomycin Double Antibody Solution, DMEM Medium, HBSS Buffer were from Gibco (USA). Serum-Free Cell Cryopreservation Solution was from Suzhou Newsaimei Biotechnology Co., Ltd (Suzhou, China). CCK-8 Kit was obtained from Guangzhou Nuolaixi Biotechnology Co., Ltd (Guangzhou, China). Sodium propionate was purchased from Chengdu Lingliu Biotechnology Co., Ltd (Chengdu, China). Indomethacin was purchased from Tianjin Xienisi Biochemical Technology Co., Ltd (Tianjin, China). Chlorpromazine Hydrochloride was from Beijing Suo Laibao Technology Co., Ltd (Beijing, China). Dio (Cell Membrane Green Fluorescent Probe) was from Shanghai Chaoneng Biotechnology Co., Ltd (Shanghai, China). Simulated Intestinal Fluid, Simulated Gastric Fluid were purchased from Shanghai Yuanye Biotechnology Co., Ltd (Shanghai, China)**.** DiR (fluorescent dye) was purchased from Guangzhou Biolight Technology Co., Ltd (Guangzhou, China). Hematoxylin-Eosin (HE) staining reagent and TUNEL apoptosis kit were obtained from Vazyme Biotech Co., Ltd (Nanjing, China). The CD31 antibody (ab182981) was purchased from Abcam (Cambridge, UK). The VEGF antibody (bs-1313r) was purchased from Beijing Biosynthesis Biotechnology Co., Ltd (Beijing, China). The mouse Interferon-gamma (IFN-γ, MM-0182M1), mouse Tumor Necrosis Factor-alpha (TNF-α, MM-0132M1), mouse Transforming Growth Factor-beta 1 (TGF-β1, MM-0135M1) ELISA kits were purchased from Jiangsu Meimian Industrial Co., Ltd (Yancheng, China). 5-Fluorouracil (5-FU, S120905) was obtained from Selleck chemicals (USA).

1.2. Characterization of ACELNs and CELNs

Spectral analysis of ACELNs

CELNs and ACELNs were added to spectral potassium bromide powder (China, Aladdin Biochemical Technology Co., Ltd.), pressed into tablets and scanned by Fourier infrared spectroscopy (Thermo Fisher Scientific Shier Technology Company), respectively. CELNs and ACELNs were ultrasonically crushed with methanol and passed through 0.22 μm organic microporous membrane. The filtrate were scanned by BlueStar A ultraviolet-visible spectrophotometer (Beijing Laibotec Instrument Co., Ltd.) and F97 Pro fluorescence spectrophotometer (Shanghai Prism Technology Co., Ltd.). CELNs and ACELNs were ground into powder, and inelastic scattered light in the wave number range of 100-3500 cm^-1^ was collected by Raman spectrometer (Renishaw inVia, Lei Nishao, UK) at the excitation wavelength of 325 nm.

# **2. Supporting tables and figures**

**Table S1** Size distribution and Zeta potential of ELNs (n = 3).

| ELNs | Hydrodynamic diameter (nm) | PDI | Zeta potential (mV) |
| --- | --- | --- | --- |
| ACELNs | 391.0 ± 9.58 | 0.287 ± 0.06 | -32.8 ± 0.62 |
| CELNs | 369.3 ± 6.24 | 0.239 ± 0.04 | -39.4 ± 1.31 |

**Table S2** IC_50_ of different cells in groups with different administration.

| ELNs | IC_50_ (mg/mL) | | | |
| --- | --- | --- | --- | --- |
|  | Lo2 | HepG2 | A549 | Caco-2 |
| ACELNs | 0.5324 | 0.1260 | 0.2584 | 0.2335 |
| CELNs | 0.1356 | 0.1453 | 0.4055 | 0.3503 |

**Table S3** Fitting parameters of release kinetic models for formononetin and calycosin released from ACELNs and B-ACELNs in PBS.

| Components | Preparations | Models | Equations | *r*^2^ |
| --- | --- | --- | --- | --- |
| formononetin | ACELNs | zero-order | Q=2.65t+43.74 | 0.5984 |
|  |  | first-order | Q=74.72(1-e^-0.54t^) | 0.9886 |
|  |  | Higuchi | Q=13.68t^1/2^+28.37 | 0.7205 |
|  |  | Korsmeyer-Peppas | Q=40.58t^0.25^(n=0.25) | 0.7867 |
|  | B-ACELNs | zero-order | Q=1.80t+79.98 | 0.4929 |
|  |  | first-order | Q=94.13(1-e^-0.94t^) | 0.8514 |
|  |  | Higuchi | Q=9.69t^1/2^+68.43 | 0.6651 |
|  |  | Korsmeyer-Peppas | Q=74.48t^0.12^(n=0.12) | 0.8066 |
| calycosin | ACELNs | zero-order | Q=4.17t+28.85 | 0.4707 |
|  |  | first-order | Q=63.41(1-e^-0.66t^) | 0.9528 |
|  |  | Higuchi | Q=18.67t^1/2^+13.19 | 0.6731 |
|  |  | Korsmeyer-Peppas | Q=31.94t^0.34^(n=0.34) | 0.7277 |
|  | B-ACELNs | zero-order | Q=1.25t+67.33 | 0.6655 |
|  |  | first-order | Q=76.01(1-e^-1.31t^) | 0.6061 |
|  |  | Higuchi | Q=6.31t^1/2^+60.31 | 0.8094 |
|  |  | Korsmeyer-Peppas | Q=64.58t^0.09^(n=0.09) | 0.9102 |

**Table S4** Fitting parameters of release kinetic models for formononetin and calycosin released from ACELNs and B-ACELNs in SIF.

| Components | Preparations | Models | Equations | *r*^2^ |
| --- | --- | --- | --- | --- |
| formononetin | ACELNs | zero-order | Q=1.48t+75.34 | 0.1808 |
|  |  | first-order | Q=88.69(1-e^-0.77t^) | 0.9725 |
|  |  | Higuchi | Q=8.66t^1/2^+64.77 | 0.3491 |
|  |  | Korsmeyer-Peppas | Q=69.44t^0.13^(n=0.13) | 0.5278 |
|  | B-ACELNs | zero-order | Q=13.71t+54.63 | 0.9657 |
|  |  | first-order | Q=106.09(1-e^-1.03t^) | 0.9859 |
|  |  | Higuchi | Q=40.14t^1/2^+28.12 | 0.9891 |
|  |  | Korsmeyer-Peppas | Q=68.24t^0.33^(n=0.33) | 0.9939 |
| calycosin | ACELNs | zero-order | Q=1.57t+68.54 | 0.3265 |
|  |  | first-order | Q=79.08(1-e^-1.19t^) | 0.8918 |
|  |  | Higuchi | Q=8.24t^1/2^+59.06 | 0.5422 |
|  |  | Korsmeyer-Peppas | Q=64.86t^0.12^(n=0.12) | 0.7135 |
|  | B-ACELNs | zero-order | Q=7.78t+101.58 | 0.6488 |
|  |  | first-order | Q=130.72(1-e^-1.23t^) | 0.9742 |
|  |  | Higuchi | Q=26.50t^1/2^+79.55 | 0.7598 |
|  |  | Korsmeyer-Peppas | Q=103.93t^0.17^(n=0.17) | 0.8302 |

**Table S5** Fitting parameters of release kinetic models for formononetin and calycosin released from ACELNs and B-ACELNs in SGF.

| Components | Preparations | Models | Equations | *r*^2^ |
| --- | --- | --- | --- | --- |
| formononetin | ACELNs | zero-order | Q=1.74t+61.74 | 0.5743 |
|  |  | first-order | Q=82.63(1-e^-0.71t^) | 0.9251 |
|  |  | Higuchi | Q=8.92t^1/2^+51.75 | 0.6385 |
|  |  | Korsmeyer-Peppas | Q=57.91t^0.14^(n=0.14) | 0.6943 |
|  | B-ACELNs | zero-order | Q=2.42t+73.00 | 0.3081 |
|  |  | first-order | Q=92.39(1-e^-1.06t^) | 0.9736 |
|  |  | Higuchi | Q=12.29t^1/2^+60.15 | 0.5090 |
|  |  | Korsmeyer-Peppas | Q=70.22t^0.15^(n=0.15) | 0.6603 |
| calycosin | ACELNs | zero-order | Q=3.32t+59.75 | 0.6375 |
|  |  | first-order | Q=80.68(1-e^-0.86t^) | 0.9546 |
|  |  | Higuchi | Q=14.14t^1/2^+46.08 | 0.7785 |
|  |  | Korsmeyer-Peppas | Q=58.15t^0.18^(n=0.18) | 0.8477 |
|  | B-ACELNs | zero-order | Q=0.59t+77.94 | 0.0887 |
|  |  | first-order | Q=81.32(1-e^-2.11^t) | 0.8193 |
|  |  | Higuchi | Q=3.24t^1/2^+74.37 | 0.0604 |
|  |  | Korsmeyer-Peppas | Q=76.51t^0.04^(n=0.04) | 0.2372 |


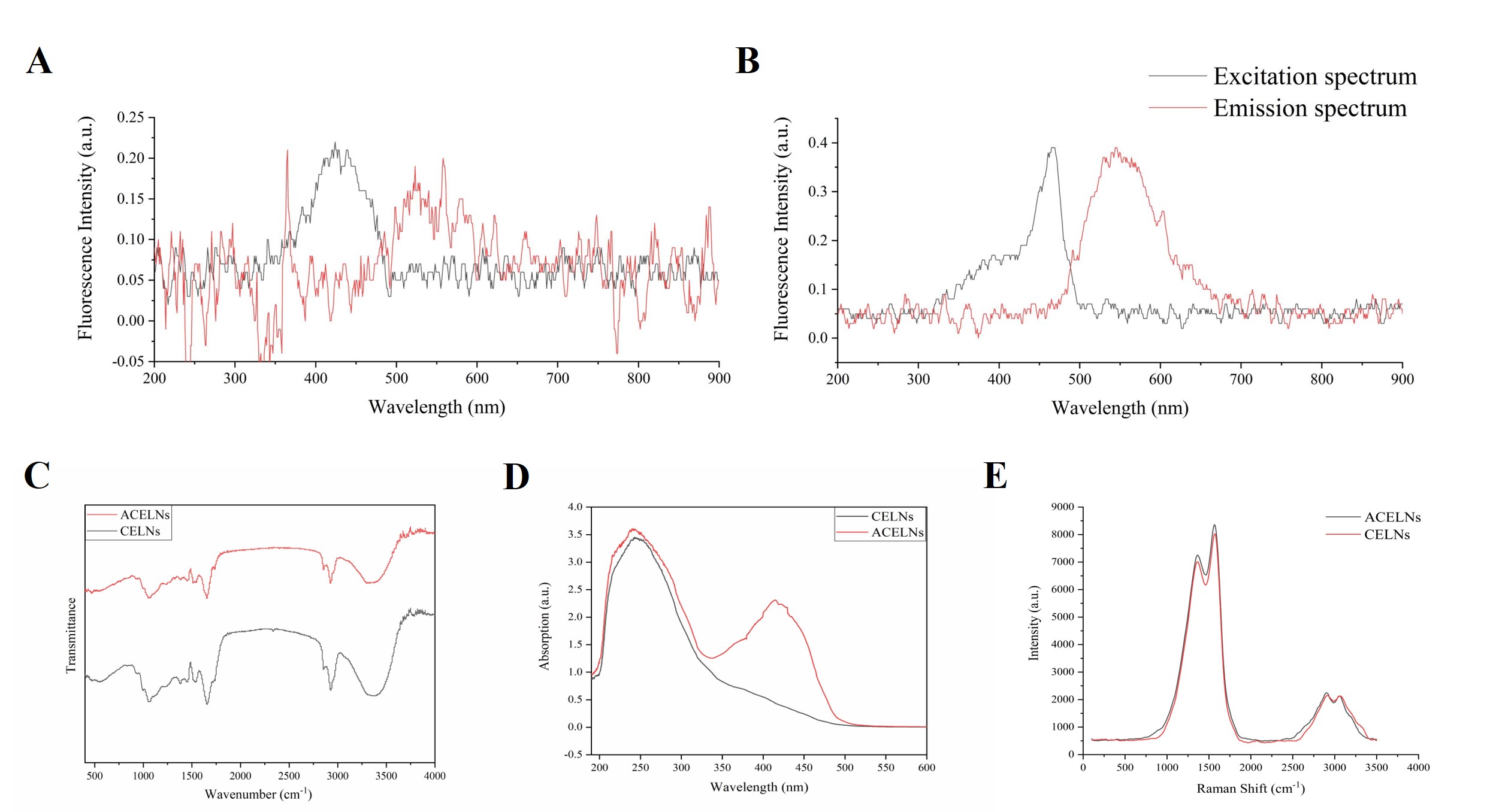


**Fig. S1.** Spectral characterization of ELNs. Fluorescence spectra of CELNs (A) and ACELNs (B), fourier transform infrared spectra (C) ultraviolet full scan spectra (D) and Raman spectra (E) of ELNs.


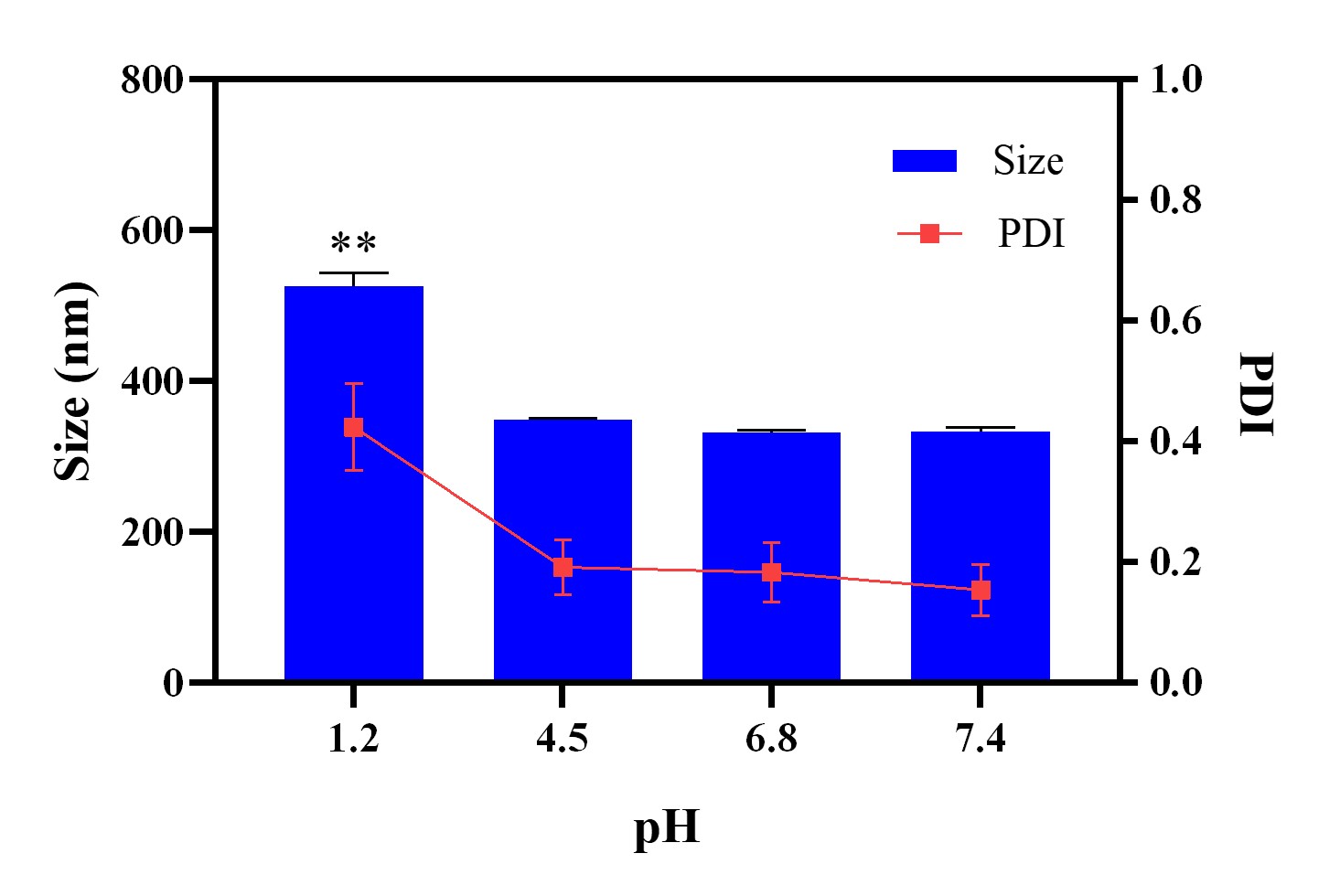


**Fig. S2.** Hydrodynamic diameter changes of ACELNs in different pH (n = 3). Compared with pH 7.4, ** *P* < 0.01.


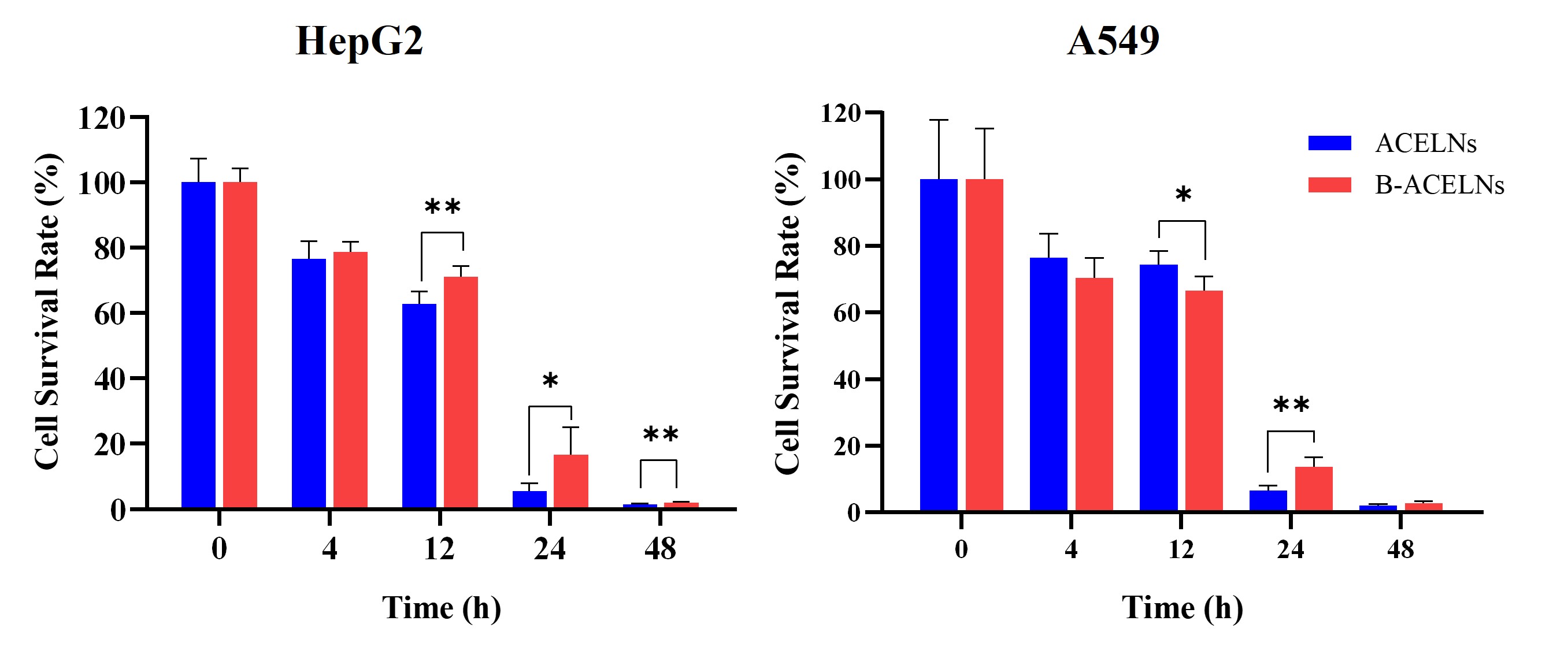


**Fig. S3.** Time-dependent cytotoxicity of ACELNs and B-ACELNs in HepG2 and A549 cells. Data are presented as mean ± SD (n = ). ACELNs compared with B-ACELNs, **P* < 0.05，***P* < 0.01.


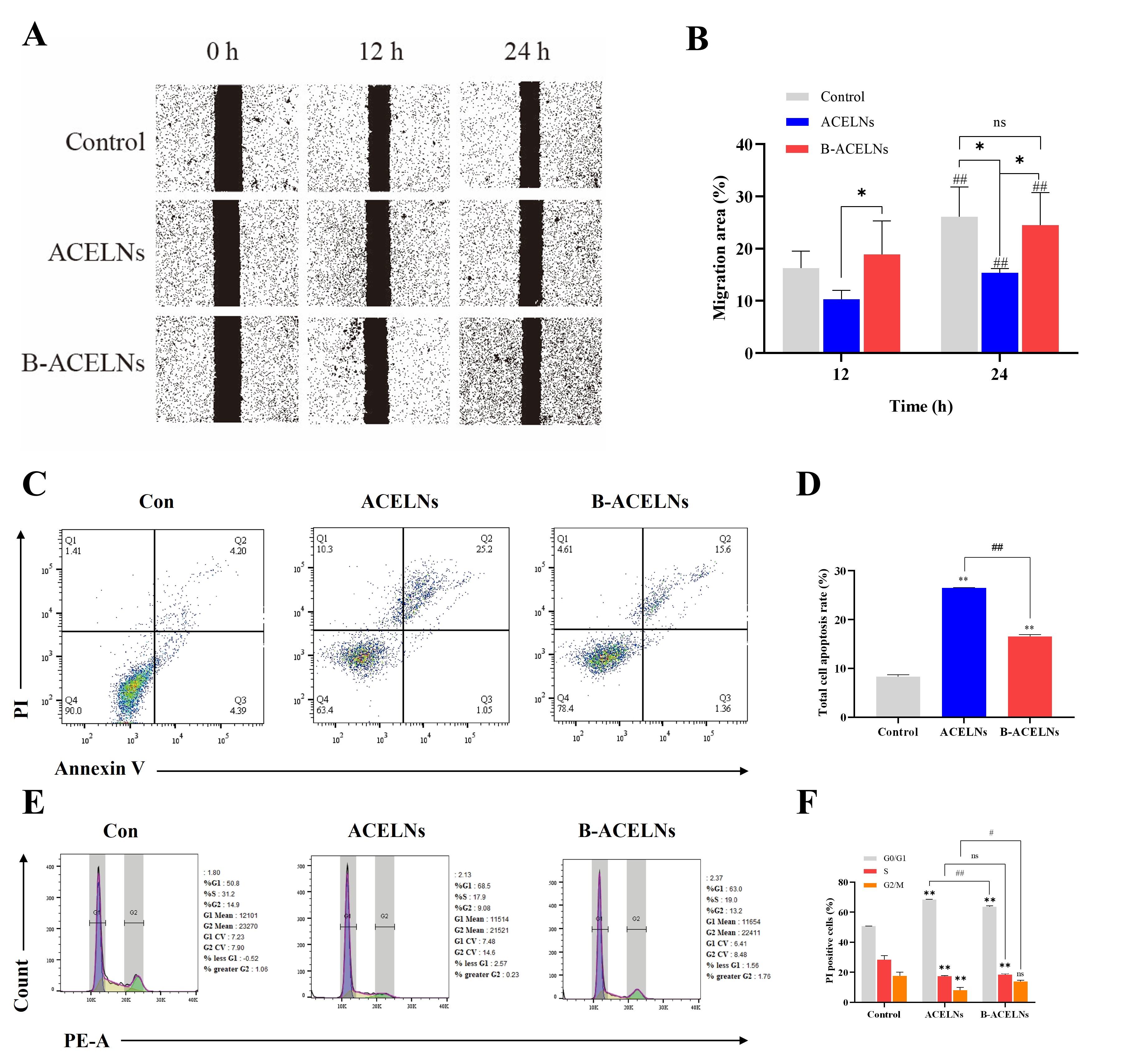


**Fig. S4.** Scratch (A, B), apoptosis (C, D) and cycle (E, F) experiment of A549 cells on ACELNs and B-ACELNs. Data are presented as mean ± SD (n = 3). Compared between groups, compared with previous time or compared with control group. **P*< 0.05, ***P* < 0.01, #*P* < 0.05, ##*P* < 0.01 and ns represents no significant difference.


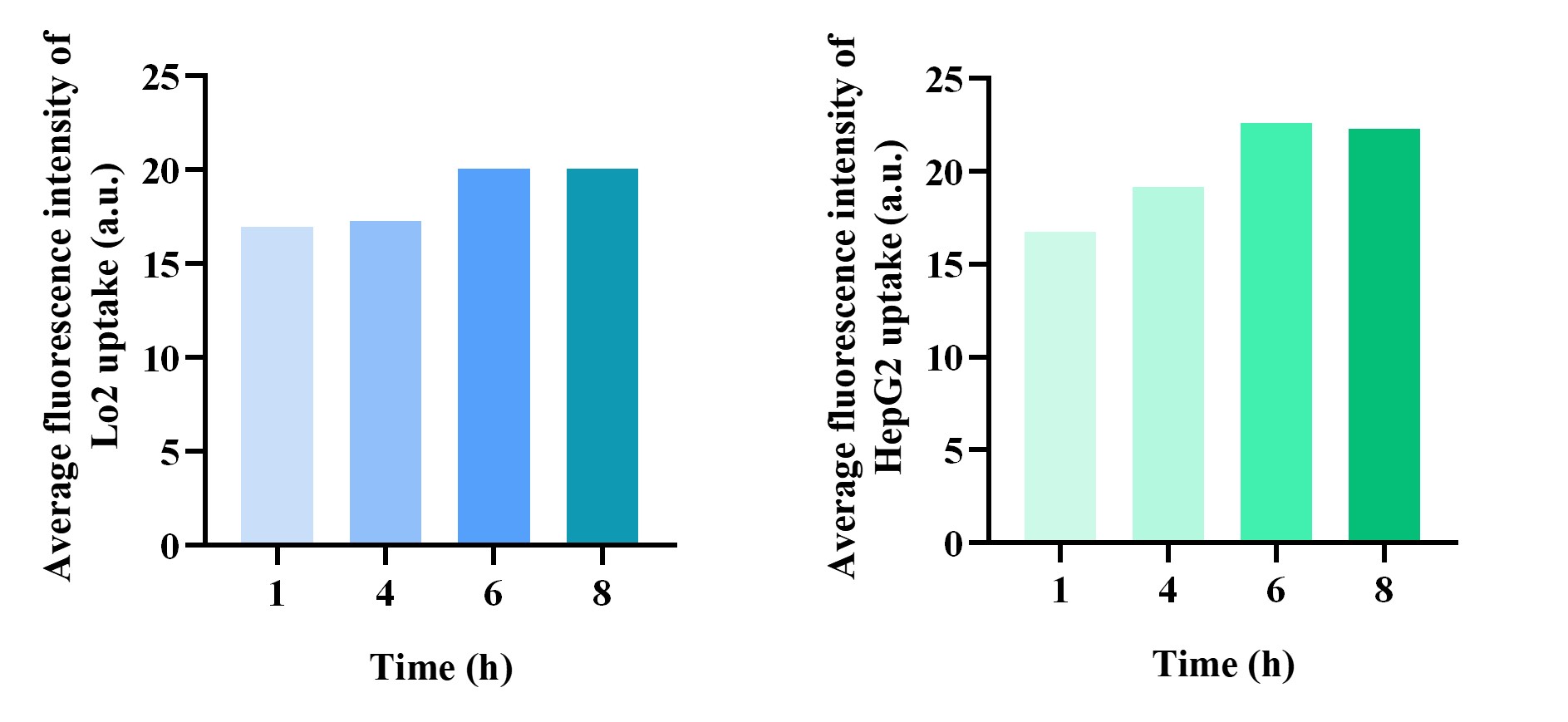


**Fig. S5.** Time-dependent cellular uptake of ACELNs. Average fluorescence intensity of ACELNs uptake in Lo2 (left) and HepG2 (right) cells at 1-8 h. Data are presented as mean ± SD (n = 3).


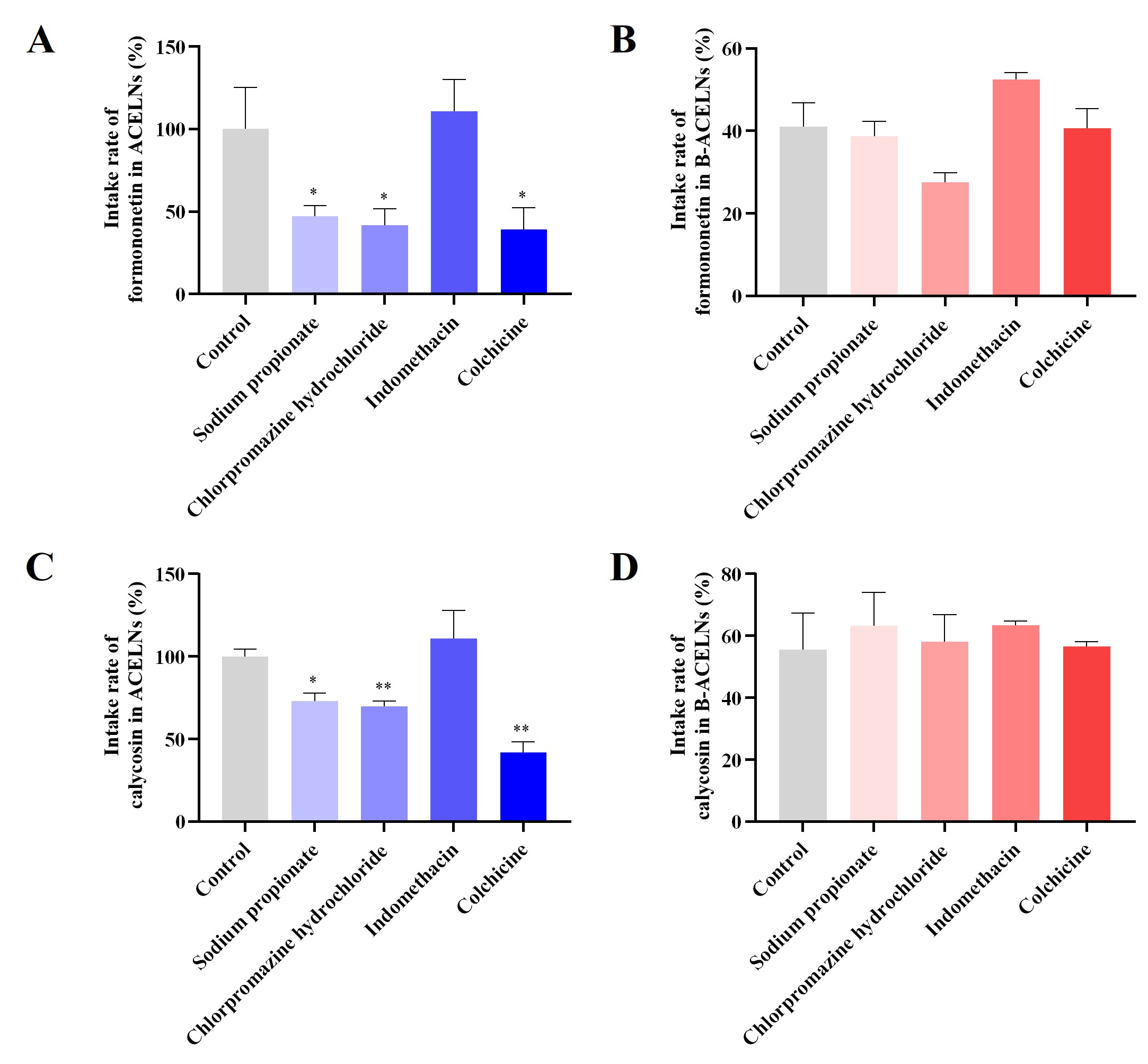


**Fig. S6.** Effects of different protein inhibitors on the uptake of active components in ACELNs or B-ACELNs by Lo2 (E, F, H, I). Data are presented as mean ± SD (n = 3). ACELNs compared with B-ACELNs or inhibitor group compared with control group, **P* < 0.05, ***P* < 0.01.


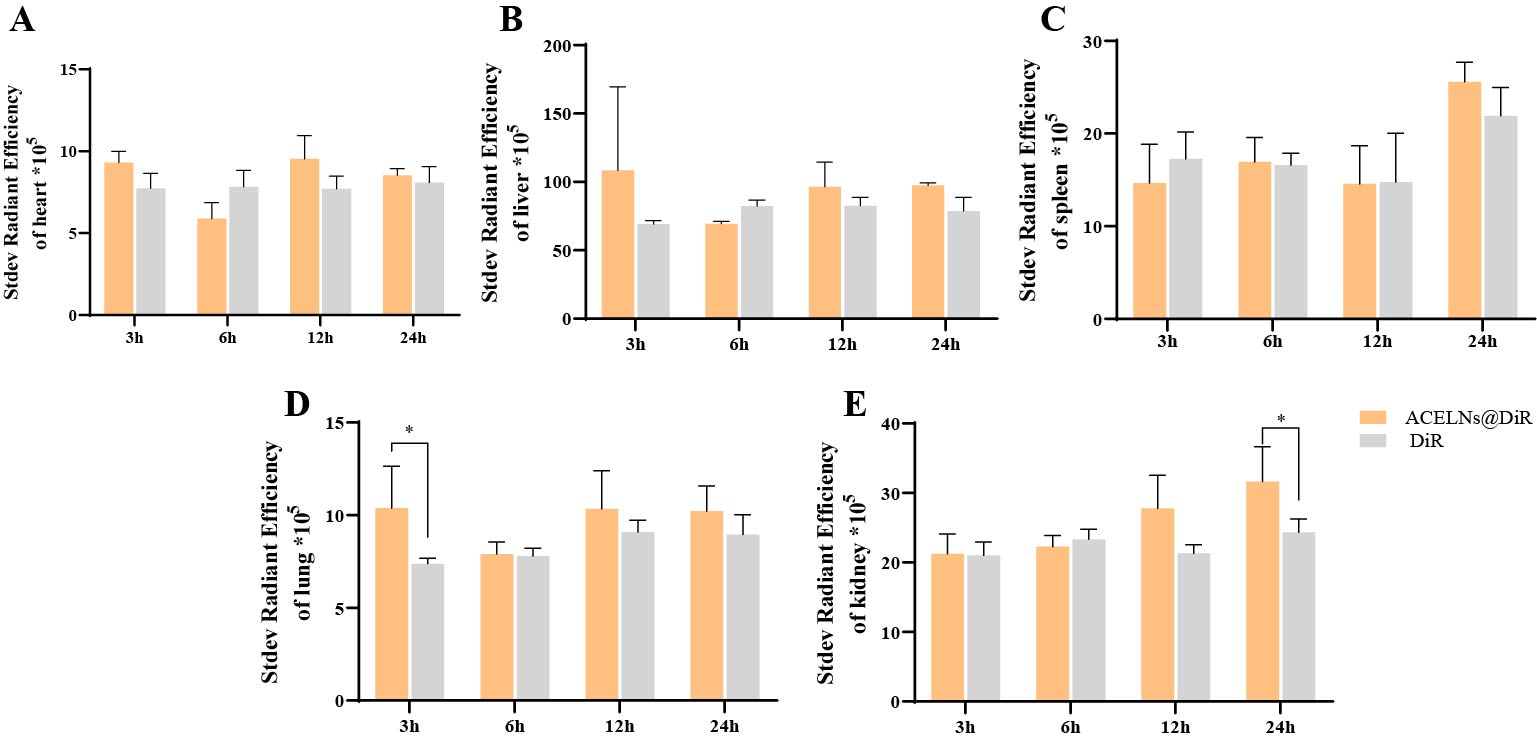


**Fig. S7.** *In vivo* distribution of ACELNs@DiR and Free DiR. Stdev radiant efficiency of ACELNs@DiR and free DiR in heart(A), liver(B), spleen(C), lung(D) and kidney (F). Data are presented as mean ± SD (n = 3). **P* < 0.05.


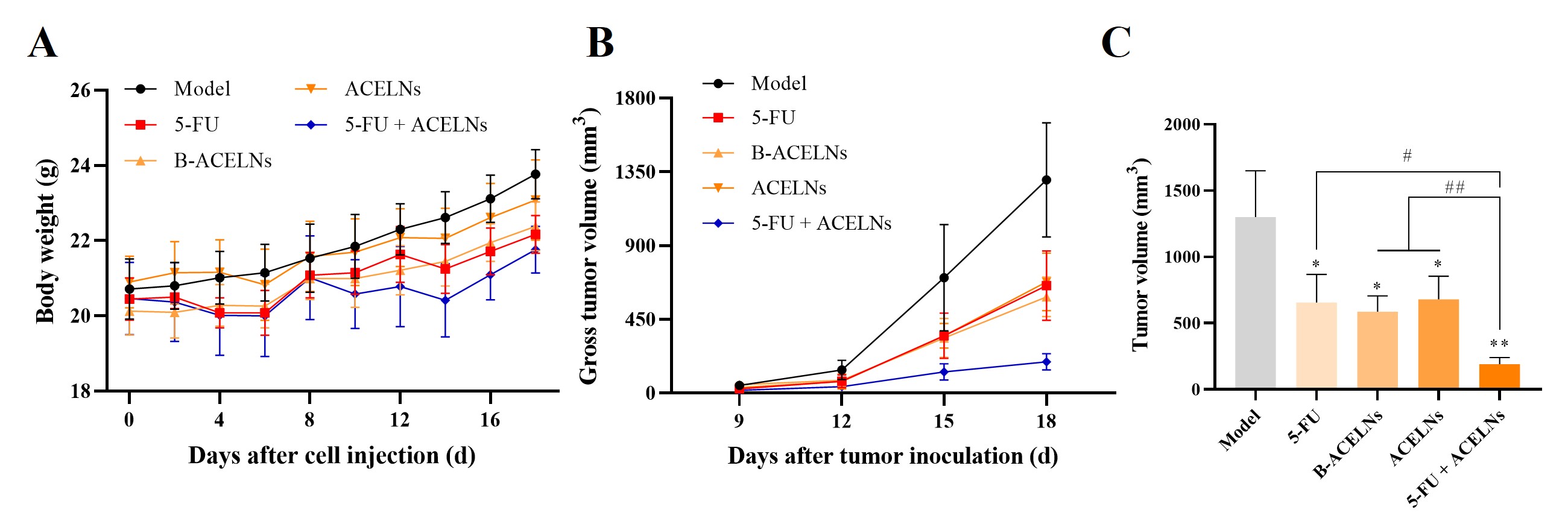


**Fig. S8.** Synergistic antitumour efficacy of ACELNs combined with 5-FU in Lewis lung cancer mice (n = 6). (A) Dynamic changes in body weight of mice during treatment. (B) Time-dependent changes in gross tumour volume. (C) Final tumour volume at the end of the experiment. Data are presented as the mean ± SD. **P* < 0.05, ** *P* < 0.01 vs. Model group; # *P* < 0.05, ## *P* < 0.01 between indicated groups.


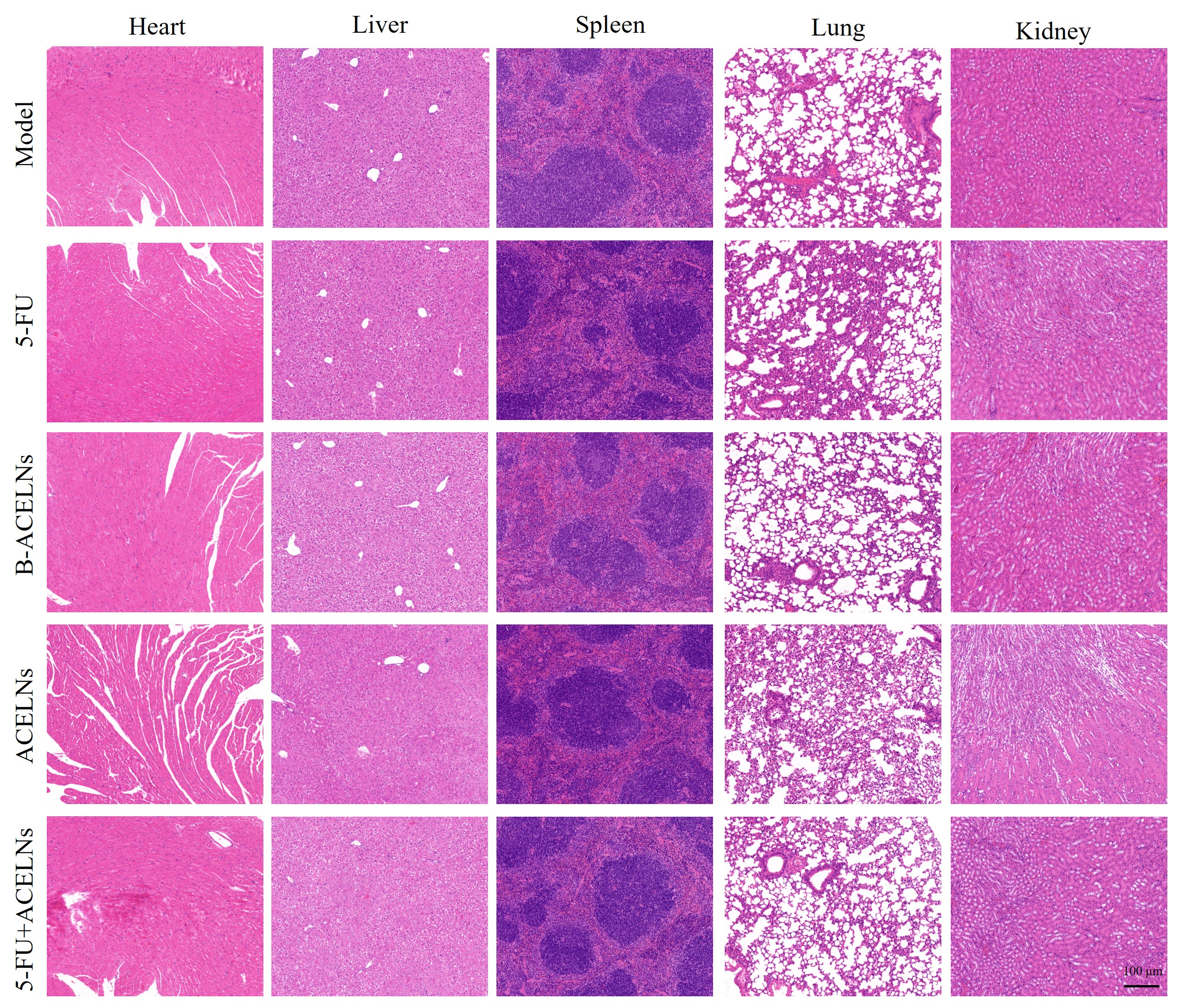


**Fig. S9.** Safety evaluation of different treatments in mice. Representative H&E staining images of heart, liver, spleen, lung, and kidney tissues (scale bar: 100 μm).


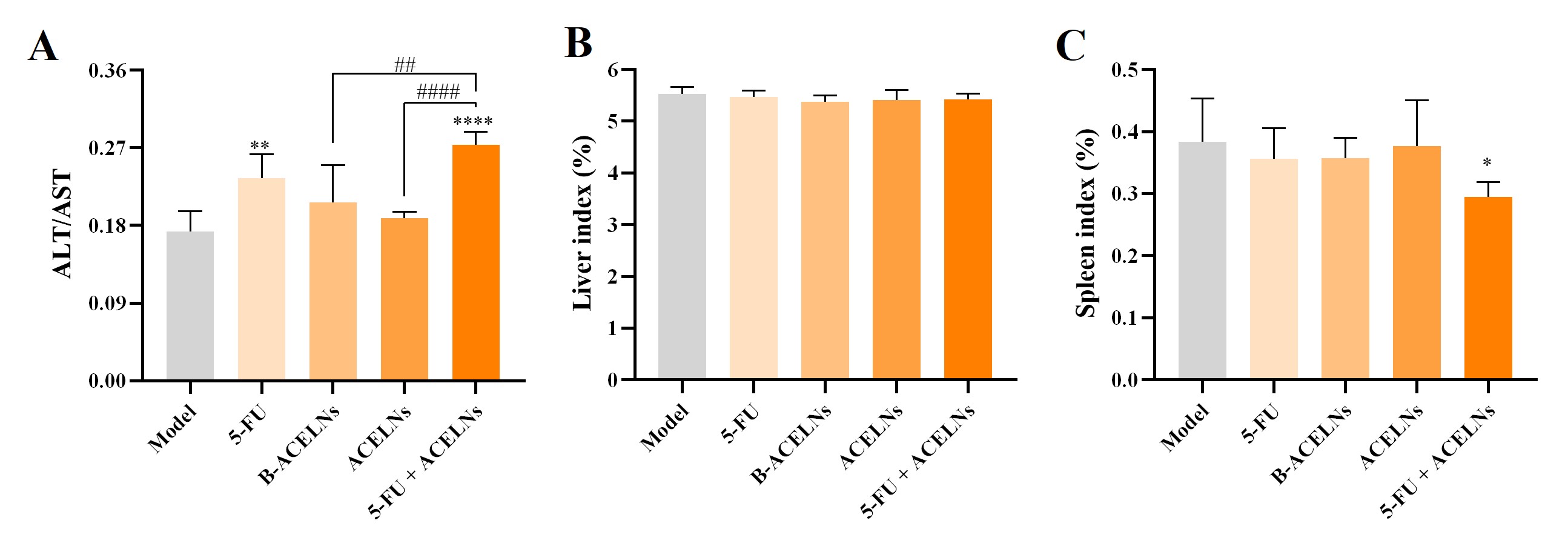


**Fig. S10.** Biochemical analysis of liver and spleen indices. (A) Ratio of ALT to AST levels in serum. (B) Liver index (organ weight/body weight × 100%). (C) Spleen index (organ weight/body weight × 100%). Data are presented as the mean ± SD (n = 6). Compared with the Model group, **P* < 0.05, ***P* < 0.01, *****P* < 0.0001; Comparison between groups, ##*P* < 0.01, ####*P* < 0.0001.


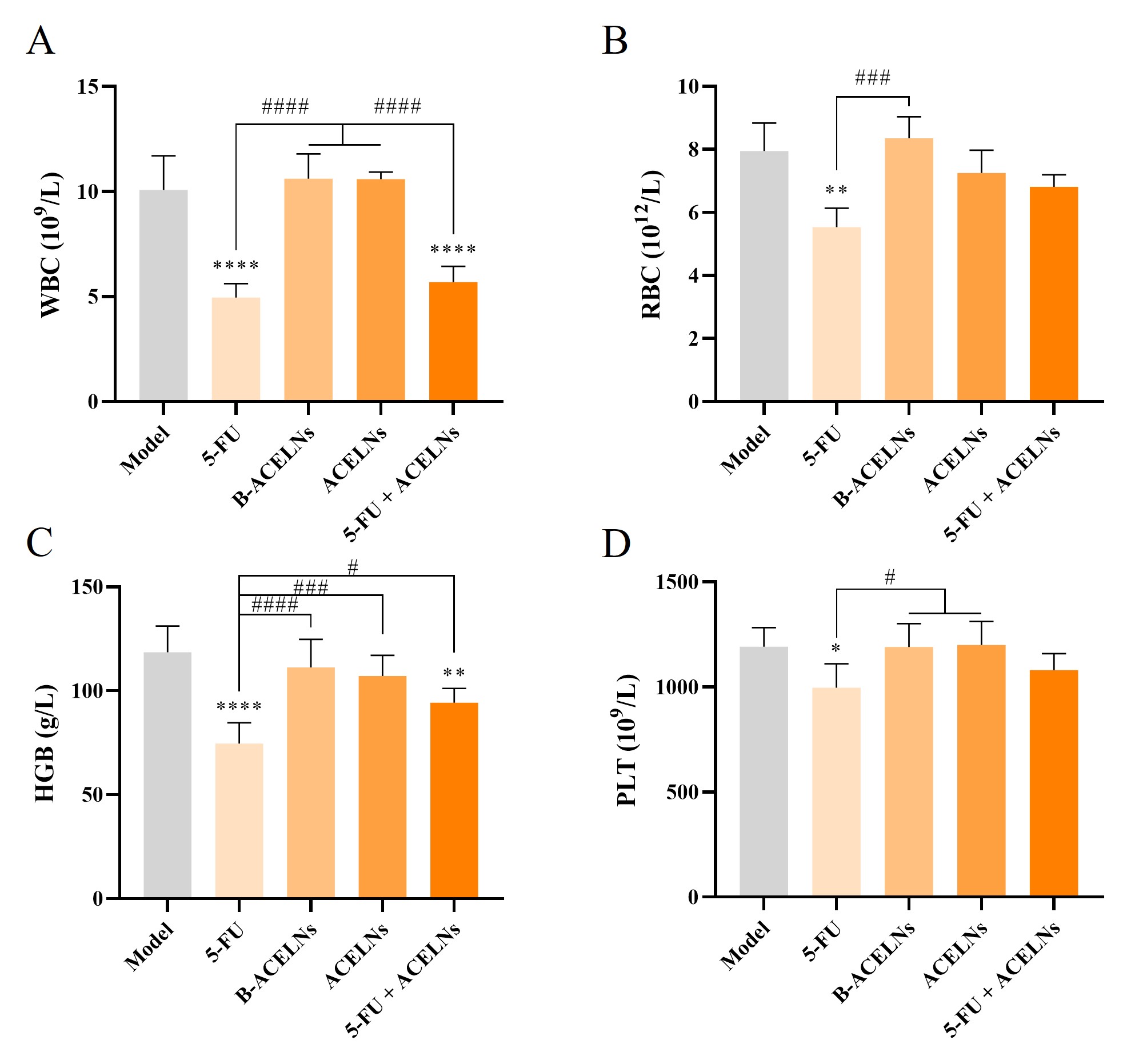


**Fig. S11.** Hematological parameters in mice from different treatment groups. (A) White blood cell count (WBC, ×10^9^/L), (B) red blood cell count (RBC, ×10^12^/L), (C) hemoglobin concentration (HGB, g/L) and (D) platelet count (PLT, ×10^9^/L) were measured to evaluate hematopoietic function and myelosuppression. Data are presented as mean ± SD (n = 6). **P* < 0.05, ***P* < 0.01, *****P* < 0.0001 denote significant differences compared with the Model group; #*P* < 0.05, ###*P* < 0.001, ####*P* < 0.0001 denote significant differences between the indicated groups.

# **3. Abbreviations**

Astragali Radix-Curcumae Rhizoma Exosome-like Nanoparticles (ACELNs)

DIR-labeled ACELNs (ACELNs@DiR)

Broken ACELNs (B-ACELNs)

Curcumae Rhizoma Exosome-like Nanoparticles (CELNs)

Exosomes-like Nanoparticles (ELNs)

Simulated Gastric Fluid (SGF)

Simulated Intestinal Fluid (SIF)

Ultra-high Performance Liquid Chromatography-tandem mass spectrometry (UPLC-MS/MS)

Dynamic Light Scattering (DLS)

Transmission Electron Microscope (TEM)

SDS-Polyacrylamide Gel Electrophoresis (SDS-PAGE)

Thin-Layer Chromatography (TLC)

Diethyl pyrocarbonate (DEPC)

Albumin from Bovine Serum (BSA)

The half maximal inhibitory concentration (IC_50_)

Penicillin-Streptomycin Solution (P/S)

Cell Counting Kit-8 (CCK-8)

Trans-Epithelial Electrical Resistance (TEER)

Apparent permeability coefficient (*P*_app_)

Fluorescein Isothiocyanate (FITC)

Propidium Iodide (PI)

Area under plasma concentration-time curve from 0 to t (*AUC*_(0-t)_)

Area under plasma concentration-time curve from 0 to infinity (*AUC*_(0-∞)_)

Mean residence time (*MRT*_(0-∞)_)

Half life of elimination (*t*_1/2z_)

Maximum plasma concentration after administration (*C*_max_)

Time when Cmax occurs (*T*_max_)

Relative bioavailability of oral drugs (*Fr*)

Alanine Aminotransferase (ALT)

Aspartate Aminotransferase (AST)

Creatinine (CREA)

Creatine Kinase (CK)

Lactate Dehydrogenase (LDH)

Enzyme-linked immunosorbent assay (ELISA)

Tumor necrosis factor-α (TNF-α)

Interferon-γ (IFN-γ)

Transforming growth factor-β1 (TGF-β1)

# **4. List of core chemical compounds**

Calycosin

Formononetin

Astragaloside IV

Curcumin

Curcumenol

β‑elemene

Furanodiene

5‑fluorouracil (5-FU)

Ginsenoside Rb1

Indomethacin
